# Enhancing mask activity in dopaminergic neurons extends lifespan in flies

**DOI:** 10.1111/acel.13493

**Published:** 2021-10-09

**Authors:** Xiaolin Tian

**Affiliations:** ^1^ Neuroscience Center of Excellence Department of Cell Biology and Anatomy Louisiana State University Health Sciences Center New Orleans Louisiana USA

**Keywords:** aging, dopaminergic neurons, mask

## Abstract

Dopaminergic neurons (DANs) are essential modulators for brain functions involving memory formation, reward processing, and decision‐making. Here I demonstrate a novel and important function of the DANs in regulating aging and longevity. Overexpressing the putative scaffolding protein Mask in two small groups of DANs in flies can significantly extend the lifespan in flies and sustain adult locomotor and fecundity at old ages. This Mask‐induced beneficial effect requires dopaminergic transmission but cannot be recapitulated by elevating dopamine production alone in the DANs. Independent activation of Gα_s_ in the same two groups of DANs via the drug‐inducible DREADD system also extends fly lifespan, further indicating the connection of specific DANs to aging control. The Mask‐induced lifespan extension appears to depend on the function of Mask to regulate microtubule (MT) stability. A structure–function analysis demonstrated that the ankyrin repeats domain in the Mask protein is both necessary for regulating MT stability (when expressed in muscles and motor neurons) and sufficient to prolong longevity (when expressed in the two groups of DANs). Furthermore, DAN‐specific overexpression of Unc‐104 or knockdown of p150^Glued^, two independent interventions previously shown to impact MT dynamics, also extends lifespan in flies. Together, these data demonstrated a novel DANs‐dependent mechanism that, upon the tuning of their MT dynamics, modulates systemic aging and longevity in flies.

## Graphical abstract

Dopaminergic neurons modulate essential brain functions in animals. In fly brains, overexpressing the putative scaffolding protein Mask in a small subset of dopaminergic neurons can significantly prolong lifespan. This effect appears to be a result of altered microtubule stability that is induced by Mask.

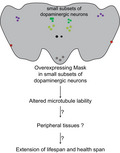

## 1 INTRODUCTION

The complex process of aging has a reciprocal interaction with the functions of the brain. This connection was indicated by studies of the neuronal‐specific genes and their ability to affect lifespan in animals (Pasyukova et al., 2015), the sensory functions of the nervous system in sensing and coordinating the responses to environmental cues that lead to impact on aging and longevity in animals (Alcedo & Kenyon, 2004; Apfeld & Kenyon, 1999; Bishop & Guarente, 2007; Chen et al., 2016; Gendron et al., 2014; Lee & Kenyon, 2009; Libert et al., 2007; Waterson et al., 2014; Zhang, Gong, et al., 2018), and the neuroendocrine system that modulates aging and longevity (Broughton et al., 2005, 2010; Satoh & Imai, 2014; Zhang et al., 2013, 2017). A recently emerging theme for the brain‐driven regulation of aging highlights the change of longevity induced by neuronal cell‐type‐specific modulations. Recent studies showed Forkhead family transcription factors that are differentially expressed in the glia and neurons each enable distinct capacities to affect longevity (Bolukbasi et al., 2021). Specific neuronal types such as the serotonergic neurons in both worms (Higuchi‐Sanabria et al., 2020; Zhang, Wu, et al., 2018) and flies (Chakraborty et al., 2019; Lyu et al., 2021; Ro et al., 2016) regulate aging through targeting different physiological functions in the peripheral tissues.

Dopaminergic neurons (DANs), a crucial modulatory system in the brain (Arias‐Carrión et al., 2010; Schultz, 2007), have also been implicated in aging regulation. Limited studies on this topic yielded controversial results: On the one hand, the function of the dopaminergic system in regulating reward‐feeding, food intake, and energy balance (Doan et al., 2016; Murray et al., 2014; Narayanan et al., 2010) implies an intuitive link to the control of aging. The results of a few previous studies support such a link: In rodents, increasing dopamine level leads to extended longevity (Cotzias et al., 1974; Knoll, 1997). In flies, polymorphism in *dopa decarboxylase* (*Ddc*), an enzyme required for dopamine biogenesis, correlates with variations in longevities in fly populations (De Luca et al., 2003). In worms, circumventing the ER unfolded protein stress in the DANs can moderately extend lifespan (Higuchi‐Sanabria et al., 2020). On the other hand, loss of DANs or dopamine production in the brain does not seem to affect longevity. Depletion of dopamine production in the fly brains (Riemensperger et al., 2011) or in worms (Murakami & Murakami, 2007) does not affect lifespans in either organism. The life expectancy of human patients with Parkinson's disease (PD) who lose their DA neurons in the substantia nigra is similar to the normal population (Savica et al., 2017). Studies in humans and rodents also showed that chronically and globally increasing dopamine levels led to adverse effects (Stansley & Yamamoto, 2015). For example, long‐term supplementation of L‐DOPA together with deprenyl at low dosages causes an increased death rate in patients at an early stage of Parkinson's disease (Lees, 1995). Therefore, it remains elusive as to whether and how the dopamine system can actively affect aging.

My studies on a putative scaffolding protein, Mask, provided new evidence supporting a function of DANs in regulating aging and longevity. Mask was previously implicated in diverse signaling pathways and cellular processes. In mitotic cells, Mask acts as a modulator of the receptor tyrosine kinase signaling in flies (Smith et al., 2002), a co‐factor of the Hippo pathway effector Yorkie (Sansores‐Garcia et al., 2013; Sidor et al., 2013) and a positive regulator of the JAK/STAT pathway (Fisher et al., 2018). Our studies of Mask in the post‐mitotic cells, such as muscles and neurons, demonstrated that it modulates mitochondrial morphology (Zhu et al., 2015), autophagy (Zhu et al., 2017), microtubule (MT) dynamics, and synaptic morphology (Martinez et al., 2021). In this study, I show that overexpressing Mask in two small groups of DANs in the fly brain significantly extends the lifespan of these flies, and this DAN‐mediated effect can be independently recapitulated by moderately activating Gα_s_ in the two groups of DANs. I also provide multiple pieces of evidence to support a model that enhanced MT dynamics in the DANs is a primary contributing factor conveying dopaminergic‐dependent lifespan extension. All these results together point to the notion that MT stability and dynamics in specific DANs are targetable processes that can be tuned to impact aging.

## 2 RESULTS

### 2.1 Small subsets of the DANs regulates longevity in flies

**FIGURE 1:**
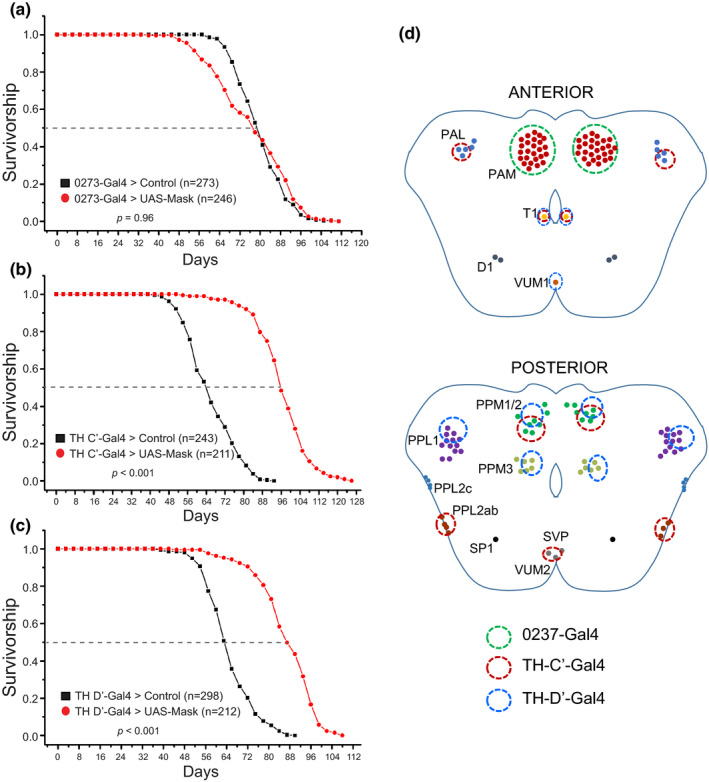
Overexpressing mask in the TH‐C’ or TH‐D’ DANs extends lifespan. Survivorship curves of male control flies and flies overexpressing Mask under the control of (a) 0273‐Gal4, (b) TH‐C’‐Gal4, and (c) TH‐D’‐Gal4 drivers. (d) Schematic of expression patterns of 0273‐, TH‐C’‐, and TH‐D’‐Gal4 drivers. Female flies are not shown but exhibit similar lifespan extensions when overexpressing Mask in the TH‐C’ or TH‐D’ dopaminergic neurons

Our previous studies on Mask uncovered diverse functions of this putative scaffolding protein in the post‐mitotic cells, including a strong protective effect against neurodegeneration induced by toxic protein aggregates (Zhu et al., 2017). In the continued investigation seeking to understand Mask's functions in neurons, I uncovered an interesting gain‐of‐function effect of Mask in the DANs. Overexpressing Mask in DANs (driven by pan‐dopaminergic Ddc‐Gal4 or TH‐Gal4 drivers) leads to an over 40% increase in lifespan in flies (median lifespan in male flies increased from ~76 days to ~104 days by Ddc‐Gal4, and from ~72 days to ~105 days by TH‐Gal4; Figures S1 and S2). Overexpressing Mask in either the entire body or the nervous system showed no effects on the lifespan (Figure S2), suggesting that other neuronal types potentially counteract the effects of DNAs on aging (Figure S3). Dopaminergic neurons in the adult fly brain cluster into eight groups (Mao & Davis, 2009), and using three well‐characterized Gal4 drivers that together show largely non‐overlapping expression in most of the dopaminergic neurons in the fly brains (Burke et al., 2012; Galili et al., 2014; Liu et al., 2012), I tested whether all or only subsets of dopaminergic neurons are responsible for the Mask‐mediated lifespan extension. Overexpressing Mask in these three groups of dopaminergic neurons yielded distinct results on longevity. Overexpressing Mask in the PAM dopaminergic neurons marked by the 0273‐Gal4 driver does not alter the lifespan (Figure 1a); however, expressing Mask driven by either the TH‐C’‐ or TH‐D’‐Gal4 drivers significantly extends the lifespan—the median lifespan in male flies increased from 64 to 96 days, and from 63 to 86 days, respectively (Figure 1b,c).

**FIGURE 2:**
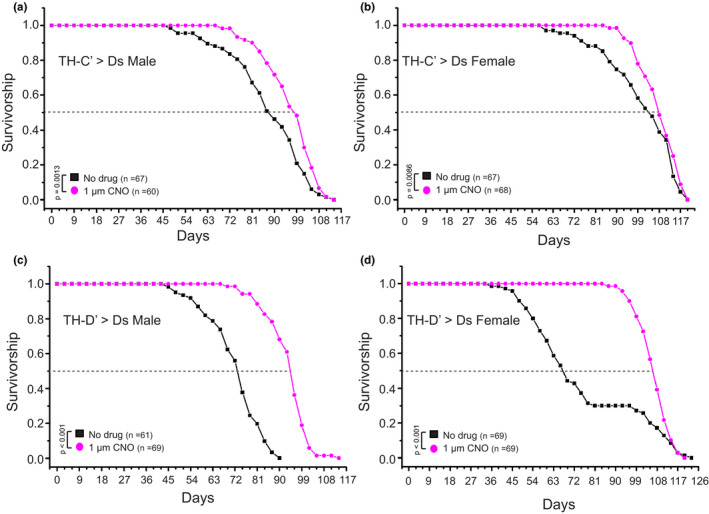
Moderately activating the ectopically expressed DREADD system in the TH‐C’ or TH‐D’ DANs extends lifespan in flies. Survivorship curves of male and female adult flies expressing the DREADD Gαs receptor and raised on food containing DMSO only (No Drug) or 1uM Clozapine N‐oxide (CNO). (a, b) moderately activating the DREADD‐Ds receptor in the TH‐C’ neurons leads to an extension of lifespan in flies. The median lifespan increased from ~87 days to ~98 days (~12.6% increase) in the male flies and from ~95 days to ~108 days (~12.6% increase) in the female flies. (c, d) moderate activation of the DREADD‐Ds receptor expressed in the TH‐D’ neurons leads to a significant extension of lifespan in flies. The median lifespan increased from ~73 days to ~95 days (~30% increase) in the male flies and from ~67 days to ~107 days (~50% increase) in the female flies

Although the dopaminergic system provides essential modulation on various behaviors and physiological functions, flies devoid of dopamine in their brains (Riemensperger et al., 2011) and worms lacking the rate‐limiting enzymes for dopamine synthesis both live a normal lifespan (Murakami & Murakami, 2007). Consistently, blocking neuronal activity in the TH‐C’ or TH‐D’ dopaminergic neurons by the DREADD‐Di (Becnel et al., 2013) does not affect the lifespan in flies (Figure S4). Together, these results suggest that dopamine systems may be dispensable for the mechanisms that drive the normal aging process. Overexpressing Mask in the specific dopaminergic neurons possibly induces a gain‐of‐function cellular effect, which consequently confers a beneficial outcome on aging and longevity. Before investigating the underlying mechanisms induced by Mask, I first set out to determine whether activation of the DANs through approaches independent of Mask overexpression could also yield similar effects on longevity. I found that continuous full activation of the TH‐C’ or TH‐D’ dopaminergic neurons via the thermosensitive cation channel TrpA1 shortened the lifespan in the male flies and had no effect on the female flies (Figure S5). However, moderate activation of these DANs induced lifespan extension in the flies (Figure 2). The inducible DREADD‐Ds receptor, when expressed in neurons, permits dose‐dependent neuronal modulation (Becnel et al., 2013). Feeding 1 μM Clozapine N‐oxide (CNO; a concentration significantly below the full activation dose (Becnel et al., 2013)) to flies expressing this engineered Gα_s_ receptor in their TH‐C’ or TH‐D’ neurons induces prominent and consistent lifespan extension (Figure 2). These results further confirm the potentials of the DANs to impact lifespan.

### 2.2 Dopamine transmission is required to elicit the Mask‐induced lifespan extension

**FIGURE 3:**
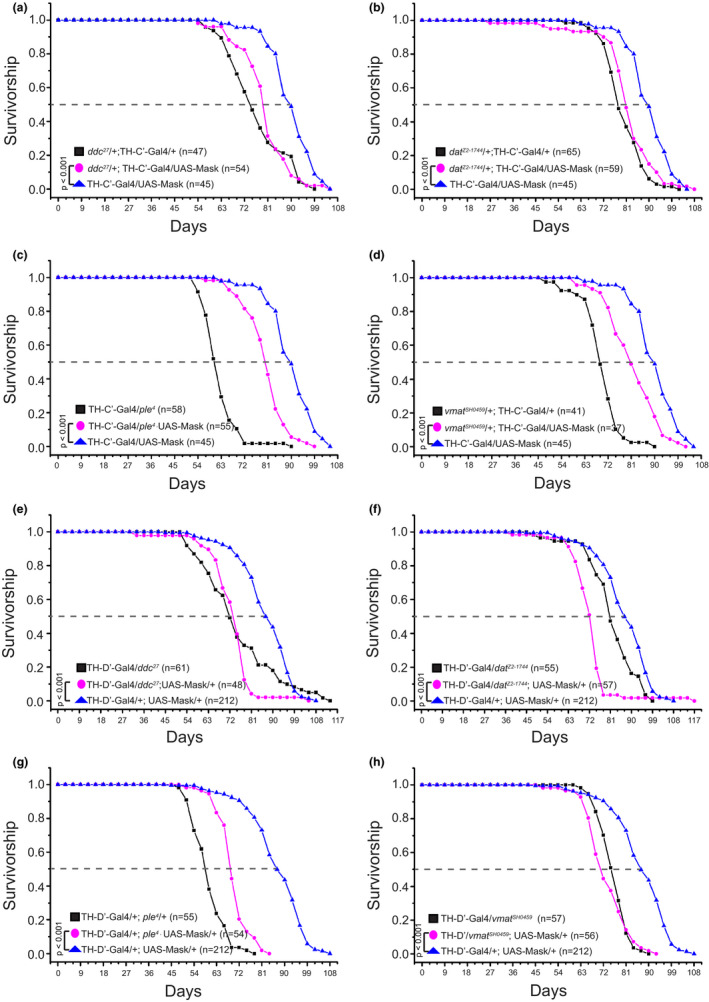
Reducing dopamine pathway activity blunts the lifespan extension effects induced by overexpressing mask in the TH‐C’ or TH‐D’ DANs. Decreasing dopaminergic pathway activity was achieved by genetically reducing the dosage of the four genes that are essential for dopamine transmission: two enzymes responsible for dopamine synthesis—Tyrosine Hydroxylase (TH) and Dopa Decarboxylase (Ddc); and two transporters for dopamine—dopamine transporter (DAT) that controls dopamine reuptake, and vesicular monoamine transporter (VMAT) that mediates synaptic vesicular loading of dopamine. Four loss of function alleles, *ple^4^* (TH), *ddc^27^*, *dat^Z2^* ^‐^ *^1744^*, or *vmat^SH0459^*, were introduced to Mask‐overexpressing paradigms to generate a heterozygous background of each gene. The survivorship curves of male flies of the following genotypes are shown: (a) Overexpressing Mask in the TH‐C’ DA neurons in wild type or in the *ddc^27^*/+ heterozygous backgrounds; (b) Overexpressing Mask in the TH‐C’ DA neurons in wild type or in the *dat^Z2^* ^‐^ *^1744^*/+ heterozygous backgrounds; (c) Overexpressing Mask in the TH‐C’ DA neurons in wild type or in the *ple^4^*/+ heterozygous backgrounds; (d) Overexpressing Mask in the TH‐C’ DA neurons in wild type or in the *vmat^SH04594^*/+ heterozygous backgrounds; (e) Overexpressing Mask in the TH‐D’ DA neurons in wild type or in the *ddc^27^*/+ heterozygous background; (f) Overexpressing Mask in the TH‐D’ DA neurons in wild type or in the *dat^Z2^* ^‐^ *^1744^*/+ heterozygous background; (g) Overexpressing Mask in the TH‐D’ DA neurons in wild type or in the *ple^4^*/+ heterozygous background; (h) Overexpressing Mask in the TH‐D’ DA neurons in wild type or in the *vmat^SH04594^*/+ heterozygous background. With all conditions, reducing gene dosage of TH, Ddc, DAT, or VMAT is able to blunt the lifespan extension induced by overexpressing Mask in DANs

The Tyrosine Hydroxylase (TH)‐positive neurons often use dopamine as the neural transmitter to communicate with other neurons but they may also co‐transmit additional transmitter (Aguilar et al., 2017; Aso et al., 2019; Granger et al., 2017). Next, I asked whether dopamine transmission is required in mediating the lifespan extension induced by Mask overexpression in the DANs. If dopamine transmission is needed, reducing dopamine signaling in these neurons would suppress the strength of the neuronal communication, which in turn may hinder the ability of Mask overexpression to extend lifespan. Four genes are known to be essential for dopamine transmission: two enzymes responsible for dopamine synthesis—Tyrosine Hydroxylase (TH) and Dopa Decarboxylase (Ddc); and two transporters for dopamine—dopamine transporter (DAT) that controls dopamine reuptake, and vesicular monoamine transporter (VMAT) that mediates synaptic vesicular loading of dopamine. Genetically heterozygosity that reduces gene dosages of either of these four genes was introduced to the Mask overexpression paradigm to test whether dopamine transmission is necessary for the lifespan extension. The results show that reducing the dosage of each of these four genes can blunt the degree of lifespan extension in flies that overexpress Mask in the TH‐C’ or TH‐D’ neurons compared with the wild‐type background (Figure 3). These results support the notion that the dopamine system is necessary for Mask to induce lifespan extension.

**FIGURE 4:**
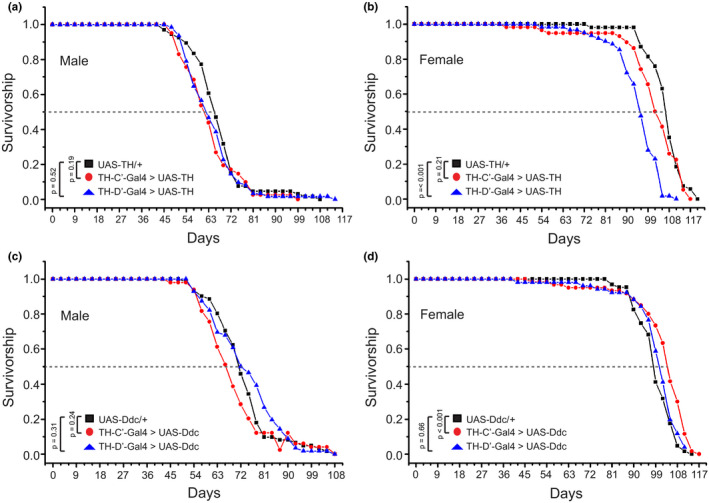
Specifically increasing dopamine production in the TH‐C’ or TH‐D’ DANs fails to extend lifespan. Survivorship curves of flies expressing the rate‐limiting enzyme for dopamine biosynthesis tyrosine hydroxylase (TH) or Dopa Decarboxylase (Ddc) in the TH‐C’ or TH‐D’ neurons are shown. The longevity of (a) male and (b) female flies overexpressing TH in the TH‐C’ or TH‐D’ DANs is recorded. This treatment does not affect the lifespan in male or female flies that overexpress TH in the TH‐C’ DANs. Female flies that overexpress TH in the TH‐D’ DA neurons show moderately reduced lifespan. (c) Male flies overexpressing Ddc in the TH‐C’ or TH‐D’ DA neurons exhibit similar lifespan compared with control flies. (d) Overexpressing Ddc in the TH‐C’ DA neurons does not affect the lifespan. However, overexpressing Ddc in the TH‐D’ DA neurons moderately extends the lifespan (median lifespan increased from 99 days in the control flies to 105 days in TH‐D’‐Gal4 > Ddc flies, ~6% increase)

I next tested whether upregulation of dopamine signaling in the TH‐C’ or TH‐D’ DANs is sufficient to induce lifespan extension in flies. In order to achieve the upregulation, the flies were fed with dopamine precursor L‐DOPA (Kayser et al., 2014), dopamine D1 receptor agonist SKF‐82958 (Walters et al., 2002), dopamine D2 receptor agonist Quinpirole (Wiemerslage et al., 2013), or the D1 and D2 agonists together. It appeared that none of the treatments was able to extend the lifespan in flies. The lifespan of female flies fed with L‐DOPA was even moderately shortened (Figure S6). This adverse effect may be attributable to the global elevation of dopaminergic signaling, as long‐term treatment with L‐DOPA has been shown to cause neural toxicity in mammals (Stansley & Yamamoto, 2015). I next limited the intervention in specific DANs. Dopamine production was selectively increased in the TH‐C’ or TH‐D’ DANs via overexpressing Tyrosine Hydroxylase (TH) or Dopa Decarboxylase (Ddc) in these two groups of neurons. Again, overexpressing these two enzymes in the TH‐C’ or TH‐D’ DANs did not prolong the longevities in flies (Figure 4). Similar to the L‐DOPA feeding, these treatments lead to a moderate reduction in the lifespan in female flies. These results together suggest that although neurons require dopamine transmission to communicate and to induce lifespan extension, increasing dopamine production or activating dopamine signaling alone is not sufficient to elicit similar effects on aging and longevity induced by Mask overexpression or moderate DREADD‐Ds activation in these neurons.

### 2.3 The long‐lived Mask‐overexpressing flies show sustained locomotor and reproduction at old ages

**FIGURE 5:**
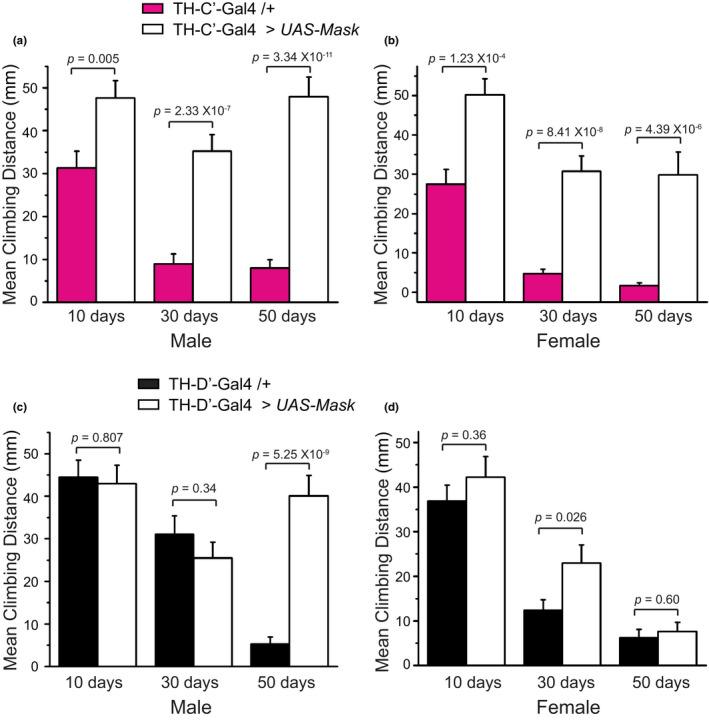
Overexpressing mask in the TH‐C’ and TH‐D’ DANs improves locomotor in aged flies. Mean climb distances in Rapid Iterative Negative Geotaxis (RING) assay were measured to assess the locomotor in adult flies. Mean climb distance of a group of 15–20 (a) male or (b) female flies at ages of day 10, 30, or 50 is shown. Flies overexpressing Mask in the TH‐C’ DANs show significantly faster climbing throughout the entire lifespan compared with the control flies. Mean climb distance of (c) male and (d) female control flies and flies overexpressing Mask in TH‐D’ DA neurons were measured at ages of day 10, 30, or 50. Flies overexpressing Mask in the TH‐D’ DA neurons are significantly more active at mid and old ages. (*n* = 3 for all sample groups). *Note*. The RING Assay performed on 10‐ and 30‐day old flies used 3 s total duration, while RING Assays on 50‐day‐old flies used 5‐s duration

To fully understand how aging and longevity at the systemic level are impacted by overexpressing Mask in the TH‐C’ or TH‐D’ neurons, I next examined age‐related phenotypes including feeding, energy storage/metabolic, adult locomotor, and brain insulin productions in Mask‐expressing and control flies. DANs regulate feeding and food‐foraging in flies (Landayan & Wolf, 2015; Liu et al., 2017; Marella et al., 2012; Tsao et al., 2018), raising the possibility that the long‐lived flies that overexpress Mask in the TH‐C’ or TH‐D’ neurons may consume less food and therefore undertake voluntary caloric restriction, which may serve as a potential contributing factor for lifespan extension in these flies. However, the Mask‐overexpressing long‐lived flies consume a comparable amount of food compared to the control flies at young and middle ages (10 and 30 days old, respectively) in the Capillary Feeder assay (CAFE assay; Diegelmann et al., 2017; Figure S7). These flies also show moderately increased whole‐body glucose and Triglyceride (TAG) levels at middle and late ages (30 and 50 days old, respectively; Figure S8). There are also no consistent alterations in transcription levels of the Dilp2, 3, 5, and 6 in their brains between genders and ages in the Mask‐expressing flies compared to the controls (Figures S9–S12). Adult locomotor reflects overall activity and health in flies and can be quantified using the rapid iterative negative geotaxis (RING) assay (Gargano et al., 2005; Nichols et al., 2012). Expressing Mask in the TH‐C’ dopaminergic neurons significantly enhances the locomotor performance during the entire lifespan, and expressing Mask in the TH‐D’ neurons sustains the adult locomotor at middle and late ages (Figure 5). Altogether, these results demonstrated that the Mask‐overexpressing flies live healthy and active lives throughout their entire lifespan.

**FIGURE 6:**
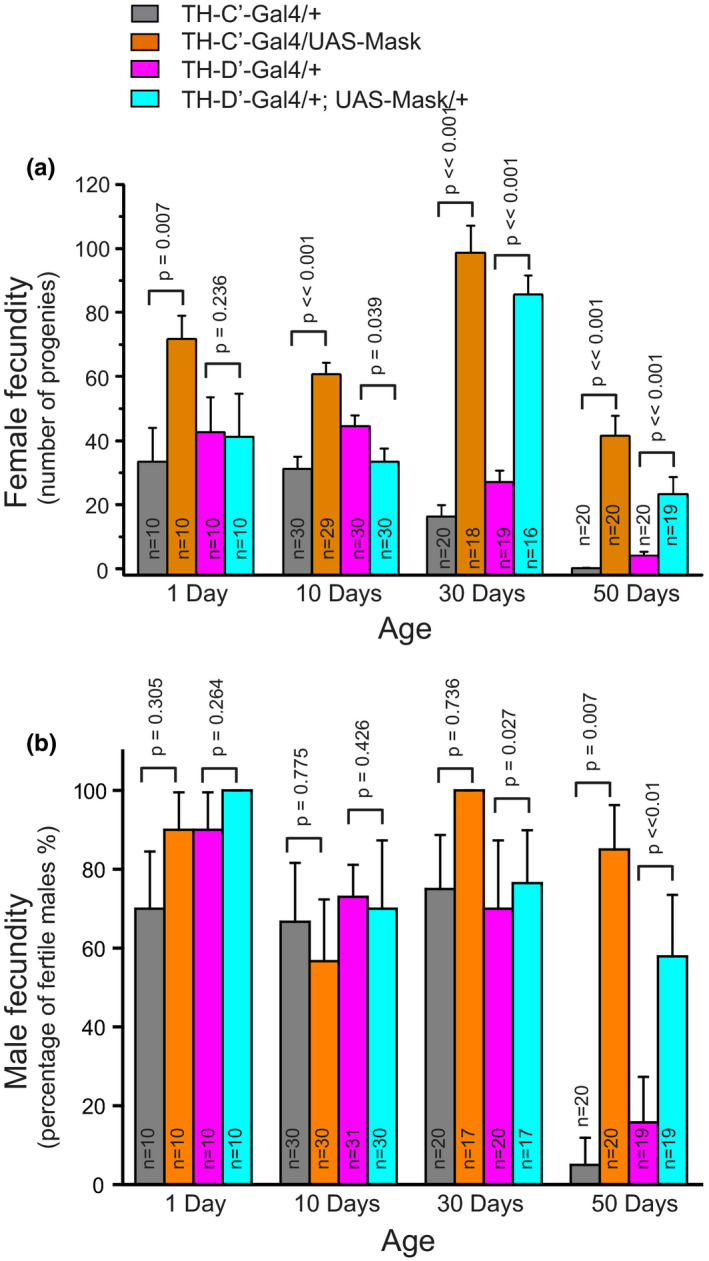
Mask‐overexpressing flies show sustained fecundity at old ages. (a) Numbers of progenies produced by female flies in a 3‐day interval. The female flies that overexpress Mask in the TH‐C’ or TH‐D’ DANs produce significantly more progenies at 30‐ and 50‐day‐old ages. (b) Reproduction in male flies is measured as the percentage of male flies that are fertile. At age 50‐day, the majority of the control male flies lose the ability to produce progeny, while over 80% of male flies that overexpress Mask in the TH‐C’ and 60% of those overexpress Mask in the TH‐D’ DANs are still reproductively active

Delayed reproductive maturation or reduced fecundity correlates closely with lifespan extension (Yuan et al., 2012). To test whether there is a tradeoff between reproduction and lifespan in Mask‐overexpressing flies, I examined the fecundity of male and female flies at different ages. No delay or reduction in reproduction in either male or female flies was detected. Instead, overexpressing Mask in the TH‐C’ neurons leads to increased fertility in female flies beginning at the onset of the reproduction maturation, and the TH‐D’‐overexpressing flies showed a significantly higher level of fecundity at older ages (Figure 6). Clearly, Mask‐overexpressing flies show a trait of extended lifespan with no cost of reproduction; and along with a number of other previous studies of longevity‐related mutant flies (Grandison et al., 2009; Hwangbo et al., 2004; Marden et al., 2003; Simon et al., 2003), this trait contradicts the long acknowledged disposable soma theory of aging.

### 2.4 Tuning microtubule (MT) stability in the DANs induces lifespan extension in flies

**FIGURE 7:**
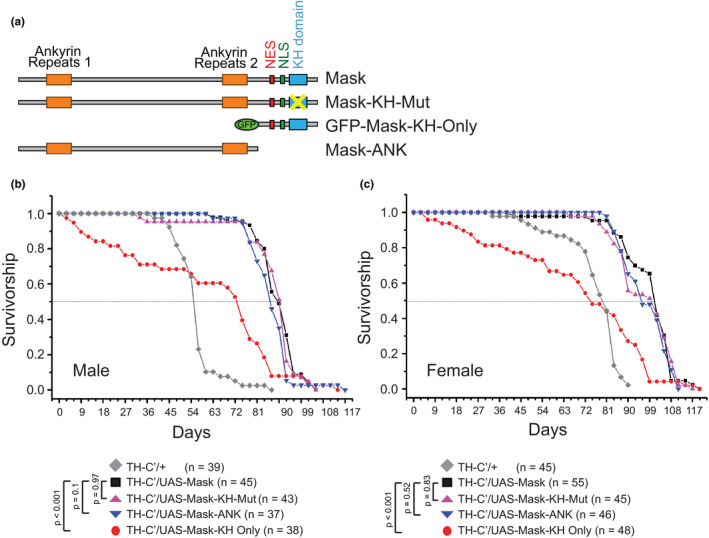
The ankyrin repeat domains in the mask protein are sufficient to induce lifespan extension. (a) Schematic of domain structures of wild‐type and Mask mutant transgenes. (b, c) Survivorship curves of male flies overexpressing different forms of Mask protein in the TH‐C’ DANs. When overexpressed in the TH‐C’ DANs, the Mask‐ANK and Mask‐KH‐Mutant transgenes show similar strength to induce lifespan extension compared with the wild‐type Mask protein

In a parallel study, we discovered that Mask promotes microtubule (MT) dynamics in fly larval motor neurons and body wall muscles (Martinez et al., 2021). I next tested whether altered MT dynamics in the Mask‐expressing DANs is the key mediator for lifespan extension. Mask is a large protein (4001 amino acids) with many functional domains. As highlighted in Figure 7, Mask/ANKHD1 bears two Ankyrin Repeats clusters, one Nuclear Export Signal (NES) and one Nuclear Localization Signal (NLS), and a C‐terminal KH domain. Ankyrin repeats mediate protein–protein interactions in eukaryotic cells (Mosavi et al., 2004). The KH domain is a conserved motif that binds RNA or single‐stranded DNA (ssDNA) and is found in proteins associated with transcription, splicing, translation, and regulation of mRNA stability (Garcia‐Mayoral et al., 2007; Grishin, 2001). In order to better understand the molecular functions of Mask, we have generated several mutant Mask transgenes (depicted in Figure 7a) to determine the structure–function correlations. One mutant transgene contains only the N‐terminal ankyrin repeats domains (Mask‐ANK); one lacks the N‐terminal half of the protein and contains the NES, NLS, and KH domains (Mask‐KH‐Only); and the third mutant transgene carries point mutations in the conserved amino acid “GXXG” motif (Hollingworth et al., 2012) in the KH domain (from GRGG to GDDG, named Mask‐KH‐Mut). A structure–function analysis performed in fly larval muscles and motor neurons revealed that the ankyrin repeats domain is necessary and sufficient to mediate the MT‐regulating function of Mask (Martinez et al., 2021), while the KH domain is required for the autophagy‐promoting function (Figure S13). Using the same set of mutant Mask transgenes, I also determined the structural requirements for Mask to induce lifespan extension. The results showed that, when expressed in the TH‐C’ DANs, the Mask‐ANK and the Mask‐KH‐Mut transgenes both have the same ability as the wild‐type Mask protein to drive lifespan extension in both male and female flies (Figure 7b,c). In contrast, the Mask‐KH only protein exhibits a dominant‐negative effect: Overexpressing this truncated protein in the TH‐C’ neurons reduces lifespan (Figure 7b,c). These results suggest that the N‐terminal portion of Mask protein containing the ankyrin repeats is necessary and sufficient to drive lifespan extension, while the NES, NLS, and the KH domains are dispensable in this context. This tight structure and function correlations between the ability of the ankyrin repeat domains to promote MT lability and to induce lifespan extension indicate that MT dynamics is one of the primary contributing factors for the DANs‐mediated beneficial effects on aging and longevity.

**FIGURE 8:**
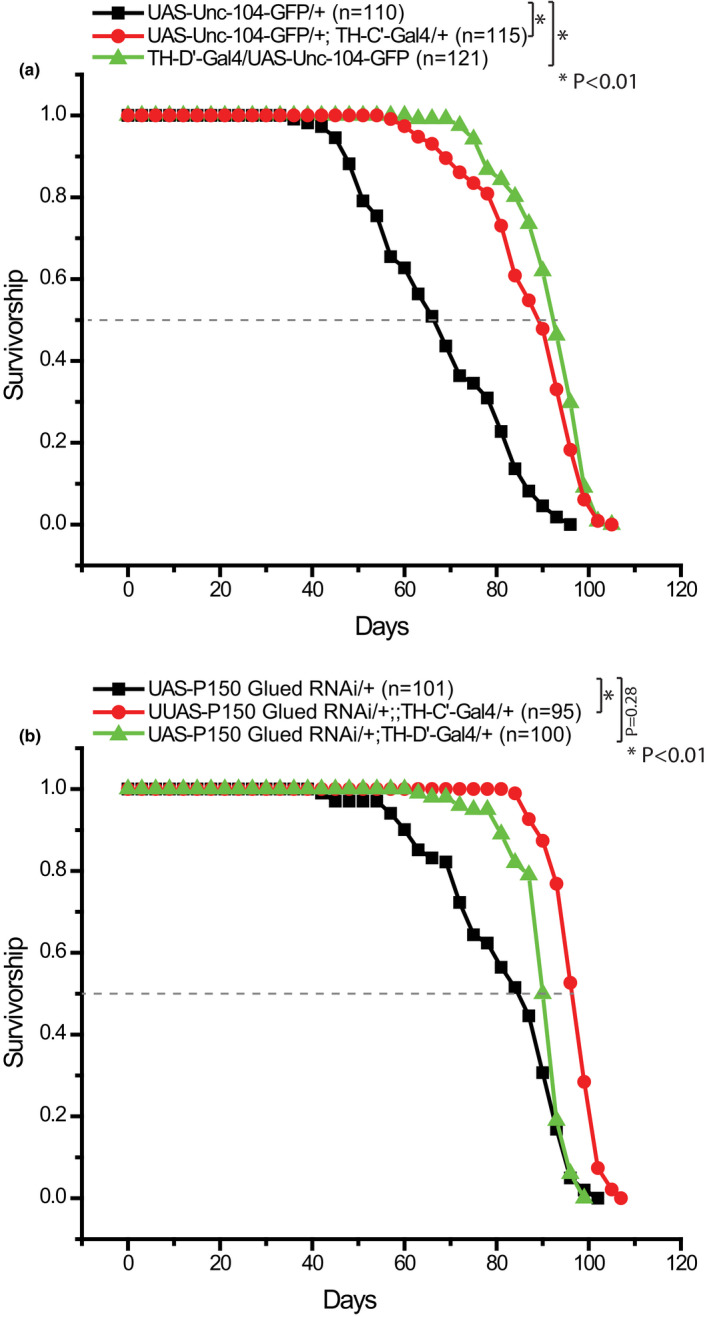
Overexpressing Unc‐104 or Knocking down p150^Glued^ in the TH‐C’ or TH‐D’ DANs extend lifespan in flies. Survivorship curves of female flies expressing UAS‐GFP‐Unc104 or a weak RNAi transgene for p150^Glued^ in the TH‐C’ or TH‐D’ DANs. (a) Overexpressing the Unc‐104‐GFP transgene in the TH‐C’ or TH‐D’ neurons leads to an extension of lifespan in flies. The median lifespan in the flies overexpressing Unc‐104‐GFP increased from ~67 days to ~92 days (~37% increase). In the flies overexpressing Unc‐104‐GFP in the TH‐D’ neurons, the median lifespan increased ~40% (median lifespan ~94 days). (b) Moderately knocking down p150^Glued^ in the TH‐C’ neurons leads to an extension of lifespan in flies, median lifespan increased from ~86 days to ~98 days (~13.95% increase). The same treatment in the TH‐D’ neurons leads to an increase of the median lifespan to ~92 days (~6.97% increase)

Based on this notion, one would expect that alternative interventions that promote MT dynamics would also be able to induce lifespan extension when applied to the same subsets of DANs. Activating the Kinesin heavy chain Unc‐104 (Randall et al., 2017; Straube et al., 2006) and knocking down a component of the Dynein/Dynactin complex, p150^Glued^ (Lazarus et al., 2013), are two independent interventions that have been previously shown to increase MT dynamics (Li et al., 2016), and overexpressing Unc‐104 in the nervous system has also been shown to moderately extend lifespan in worms (Li et al., 2016). In flies, the lifespan was extended by ~37% when Unc‐104 is overexpressed in the TH‐C’ DANs, and ~40% in the TH‐D’ DANs (Figure 8a). Reducing p150^Glued^ levels in the DANs exhibited similar but milder effects only when it is applied to the TH‐C’ DANs—the lifespan was extended by ~14%. These results together further demonstrate that increasing MT dynamics in the TH‐C’ or TH‐D’ DANs is sufficient to induce lifespan extension in flies.

## 3 DISCUSSION

In this study, I demonstrated that overexpressing the putative scaffolding protein Mask in two small groups of the DANs has a profound effect on longevity. Mask overexpression likely causes a gain‐of‐function effect that impacts the state of MT stability in these DANs. This effect would then induce a range of cellular changes that ultimately lead to altered neuronal outputs that can be relayed to the peripheral tissues, resulting in improved aging and prolonged lifespan. These findings uncovered a novel mechanism that modulates aging, but in order to fully understand this mechanism, many questions need to be answered. Among those, the most critical ones are: what are the peripheral tissues/organs that play a primary role to convey the impact on aging imposed by specific DANs; and how and through what neuronal circuits do these DANs signal to the peripheral tissues?

The DANs are composed of a heterogeneous group of neurons with diverse morphology and functions (Vogt Weisenhorn et al., 2016) that regulate a range of key brain activities. This work demonstrated that specific small subsets of DANs can also regulate aging and longevity in flies. How might these small groups of DANs modulate systematic aging? One of the well‐established DAN functions is the control of reward‐feeding and energy balance at the whole animal level (Doan et al., 2016; Murray et al., 2014; Narayanan et al., 2010). However, it is unclear whether DANs affect aging through the regulation of feeding and metabolism. Our analysis on the Mask‐overexpressing long‐lived flies indicated that the TH‐C’ and TH‐D’ DANs do not directly affect the amount of food consumption (Figure S6). At the same time, the whole‐body TAG levels in these flies are elevated (Figure S7), instead of being depleted as was indicated in the studies in worms that DAN‐mediated regulation of lipid homeostasis underlies the related lifespan extension (Higuchi‐Sanabria et al., 2020). The TAG data, together with the sustained adult locomotor (Figure 5), thus may reflect an extended healthspan in those long‐lived Mask‐overexpressing flies. Therefore, diet and metabolism are unlikely to be the primary underlying mechanism for the Mask‐induced DAN‐dependent lifespan extension. The Mask‐induced DAN‐dependent regulation on aging is also likely independent of disruptions in insulin production because the long‐lived flies showed no consistent and relatable reduction of Dilp transcript levels in their brain (Figures S9–S12). This result is somewhat consistent with the finding that disruptions of the insulin pathway in worms enhance lifespan but have no effect on the age‐dependent decline of dopamine levels (Yin et al., 2014).

It is intriguing that the prolonged lifespan of the Mask‐overexpressing flies is accompanied by a sustained fecundity in the long‐lived flies (Figure 6). Similar phenomena were also previously discovered by a number of other studies (Grandison et al., 2009; Hwangbo et al., 2004; Marden et al., 2003; Simon et al., 2003). Moreover, the reproductive female of eusocial insects shows acquirable expansion of reproduction and lifespan. Together, these phenomena challenge the long‐noticed theory about the tradeoff between reproduction and longevity, and suggest that a mechanism capable of extending both reproduction and longevity may exist as a common strategy for animals to actively intervene the aging process in order to cope with their reproductive demand. DANs, as critical modulators for brain functions, may be a vital part of a mechanism coordinating the sustainment and fulfillment of reproduction in animals. Future investigations that delineate the roles of DANs in regulating aging and reproduction can provide insight into the molecular and cellular basis for such a mechanism.

Mask and its mammalian homologs have been shown to play diverse functions in cells, including signal transduction (Fisher et al., 2018; Smith et al., 2002), transcription (Sansores‐Garcia et al., 2013; Sidor et al., 2013), autophagy (Zhu et al., 2017), and vesiculation (Kitamata et al., 2019). Our most recent study demonstrated that Mask is also a potent regulator of MT stability (in press). Which of these functions contribute to Mask‐induced lifespan extension? The structure–function analysis of the functional domains of Mask protein and our analysis of Mask‐dependent manipulation of MT stability (Figure 7) provided strong evidence suggesting that Mask's ability to promote MT dynamics is a primary contributor to the DAN‐mediated lifespan extension. This notion is further supported by the similar ability of Unc‐104 overexpression or p150^Glued^ knockdown in the DANs to extend lifespan. It is also worth noting that activation of Gα_s_ was shown to enhance MT dynamics (Sierra‐Fonseca et al., 2017), suggesting that the ability of moderately activating the engineered Gα_s_ receptor DREADD‐Ds in the DANs to extend lifespan may share similar cellular mechanism as Mask overexpression. Furthermore, I performed additional analysis to determine whether Mask's actions in regulating autophagy or vesiculation contribute to its ability to induce the dopamine‐dependent lifespan extension. First, the Mask transgene carrying the KH domain mutation fails to enhance autophagy (Figure S13) but is sufficient to induce lifespan extension. Overexpressing ATG‐1, a potent inducer of autophagy, in all neurons in the adult flies can extend lifespan (Ulgherait et al., 2014); however, overexpressing Mask in all neurons in the adult flies failed to induce similar effects (Figure S14). These data argue against the contribution of enhanced autophagy to the Mask‐induced lifespan extension. Second, I found that expressing in motor neurons increases the size of the presynaptic Rab5‐positive vesicle pools (Figure S15) and vesicular recycling (measured by FM‐143 dye loading assay, see Figure S16) at the larval NMJs, consistent with the studies of the mammalian Mask homologs in promoting vesiculation (Kitamata et al., 2019). However, such action of Mask does not seem to be sufficient to induce lifespan extension. Overexpressing the active form of Rab5, which has been shown to enhance synaptic vesicle fusion efficacy and firing strength (Wucherpfennig et al., 2003), in the DANs could only moderately extend lifespan in the male flies, with a level that is much less significant compared with the Mask‐induced lifespan extension (Figure S17). Thus, although Mask can enhance endocytosis and vesicle recycling at the nerve terminal, promoting these events alone in the DANs is not sufficient to induce the same outcome in lifespan extension as the Mask overexpression.

The MT network provides a structural platform for many essential cellular functions. The integrity of MTs is particularly important for neurons both during development and in adulthood for maintaining their normal activities. The stability and lability of the MTs are subjective to tight regulations in response to neuronal activities and stresses. Both normal and diseased aging of the brain is linked to the dysregulations of MT dynamics in neurons (Baas et al., 2016). It has been shown that DA neurons are more vulnerable to perturbation in MT stability (Ren et al., 2005), although it is currently unclear what molecular basis underlies such a distinct property of the DANs. Given the broad contributions of the MT network to the integrity of neuronal structure and function, it is conceivable that enhancing the dynamics of the MT network in neurons could induce a wide range of cellular changes. These cellular changes could collectively alter neuronal behaviors and their circuitry outputs, improve their cellular homeostasis, and mitigate the adverse effects that occur over age. The DANs, with their peculiar morphology, activity, metabolism, and molecular signature, may be more sensitive to fine tunes of MT stability and dynamics. Therefore, the lifespan extension induced by overexpressing Mask in the specific DANs can be attributed to a combination of both the abilities of these DANs to regulate the functions of specific peripheral tissues and the DANs’ intrinsic properties that allows them to better respond to Mask activity. It is unclear whether the expression levels of Mask or the molecular events controlled by Mask activity show age‐dependent alterations in the brain, or particularly in the DANs. Answers to these questions may provide a better understanding of the mechanisms underlying the lifespan extension induced by DAN‐specific expression of Mask.

## 4 METHOD AND MATERIAL

### 4.1 Drosophila strains and genetics

Flies were maintained at 25°C on standard food containing agar (0.75% w/v), cornmeal (5% w/v), molasses (4.15% v/v), and yeast (1.575% w/v) at 25 °C in a 12:12 light:dark cycle. The following strains were used in this study: UAS‐Mask (Zhu et al., 2015), TH‐C’‐Gal4 (Liu et al., 2012), TH‐D’‐Gal4 (Liu et al., 2012; from Dr. Mark Wu)_,_ 0273‐Gal4 (from Dr. Thomas R. Clandinin), UAS‐DREADD‐D_s_ (Becnel et al., 2013; from Charles Nichols), Ddc‐Gal4 (Bloomington Stock #7010); UAS‐unc104‐GFP (Bloomington Stock #24787); UAS‐p150^Glued^ RNAi (Bloomington Stock #24761). Control flies used in the study are *w^1118^* from BestGene Inc. in which the UAS‐Mask line was generated (Zhu et al., 2015) and Iso (Murakami & Murakami, 2007; from Dr. Mark Wu) in which TH‐C’‐Gal4 and TH‐D’‐Gal4 flies were generated (Liu et al., 2012).

### 4.2 Fly longevity assay

The procedures are adapted from the previously described protocol (Linford et al., 2013). 15–20 male or female flies were separated into one vial, and a total of 4–6 vials of flies were used for each genotype for longevity measurement. The flies are maintained at 25°C on standard food or food containing drugs and are transferred into fresh vials every 3 days.

### 4.3 DREADD activation

10 mM Clozapine N‐oxide (CNO; Tocris 4936) stock solution was prepared in DMSO. Food containing control (DMSO only) or 1 µM CNO was prepared freshly every three days before the flies were transferred to new vials during the longevity recording experiments.

### 4.4 Fly fecundity assay

Newly eclosed individual virgin male or female flies (day1) were crossed with 2–3 *w^1118^* virgin female or male flies respectively and kept together for 3 days. The total progeny produced within the 3‐day intervals were counted after they eclose as adult flies. Sexually exposed and active male or female flies reared together with *w^1118^* female or male were aged till day 10, 30 and 50. Each female fly was then separated into a single vial and allow to lay eggs for 3 days. The total progeny produced within the 3‐day intervals were counted after they eclose. The aged male flies were individually mated with three 5‐ to 7‐day‐old *w^1118^* virgin females for 3 days, and fecundity was measured as the percentage of males that can successfully produce progenies.

### 4.5 Statistical analysis

Data analysis for longevity: The Kaplan–Meier estimator was used to analyze the data and the survivorship curves were generated in Origin. The log‐rank test was performed with Evan's A/B tools.

Each sample was compared with other samples in the group (more than two) using one‐way ANOVA, or with the other sample in a group of two using a *t* test. The graphs were generated in Origin (Origin Lab).

## CONFLICT OF INTEREST

The author certifies that she has NO affiliations with or involvement in any organization or entity with any financial interest, or non‐financial interest in the subject matter or materials discussed in this manuscript.

## AUTHOR CONTRIBUTIONS

X.T. is responsible for experimental design, data collection, and analysis. X.T. prepared the manuscript.

### OPEN RESEARCH BADGES

This article has earned an Open Data Badge for making publicly available the digitally‐shareable data necessary to reproduce the reported results. The data is available at https://www.biorxiv.org/content/10.1101/2020.06.15.153056v2.

## Supporting information

Supplementary Material

## Data Availability

The data that support the findings of this study are openly available in BioRxiv at https://www.biorxiv.org/content/10.1101/2020.06.15.153056v2
