# Supplementary material for: Enhancing mask activity in dopaminergic neurons extends lifespan in flies
Source: Aging Cell. 2021 Oct 9;20(11):e13493. doi: 10.1111/acel.13493 (PMC8590106; doi:10.1111/acel.13493)
Supplement: Supplementary file 1 — Supplementary Material [file ACEL-20-e13493-s001.pdf]

Supplemental Information

SI Methods and Material

Fly stocks

The following stocks were used: UAS-*mask* RNAi[1], elav-Gal4[2] (neuron specific), Repo-Gal4 (glial specific); DA-Gal4 (Ubiquitous), UAS-DREADD-Di (from Charles Nichols), UAS-MASK-KH-Mutant (this study). Stocks from the Bloomington stock center include UAS-Ple or UAS-TH (37539), UAS-Ddc (37540), UAS-tdTomato (64746), UAS-TrpA1 (26263), ple^{4} (3279), ddc^{27} (3190), $dat^{Z2\text{-}1744}$ (30867), $vmat^{SH0459}$ (29477), UAS-GFP-mCherry-ATG8 (37749), GS-elav (43642), UAS-ATG1 (51654), UAS-YFP-Rab5 (9775), UAS-YFP-Rab5-S43N (9771), UAS-YFP-Rab5-Q88L (9774).

Drug Treatment

Dopamine pathway activation: microwave melted pre-made standard fly food was blended with drugs or the same amount of water or their solvent. The final concentrations for the drugs were used in this study: 1mg/ml L-DOPA[3,4] (3mg/ml L-DOPA stock in water, 0.1% L-ascorbic acid to prevent oxidation of L-DOPA); 30uM Quinpirole[5] (30mM Quinpirole stock was prepared in water); and 75uM SKF-82958[6] (15mM SKF-82958 stock was prepared in water).

Gene Switch system: Flies were raised on RU-486 (50ug/ml) food for the entire lifespan, control group were kept on food added equal amount of ethanol (10mg/ml RU486 stock was dissolved in ethanol).

Generation of UAS-Mask mutant Transgenes

The full-length wild type *mask* cDNA[1] was used to generate mutant and truncated *mask* cDNA, including pUAST-Mask-KH-Mut, pUAST-GFP-Mask-KH-Only, pUAST-Mask-Ank. To generate the UAS-Mask-KH-Mut transgenes, a complementary primer pair containing the mutated coding sequence (GGA<u>GACGAT</u>GGA, mutations underlined) were used to facilitate the replacement of the amino acid sequence (AA3053-3056) from GRGG in the wild type Mask to GDDG in the Mask-KH-Mut transgene. We used the QuickChange II XL site-mutagenesis kit (from Agilent Technology, Santa Clara, CA) to substitute wild type sequence with the mutant sequence. The entire UAS-Mask-KH-Mut coding region was then sequenced to ensure no unintended mutations were introduced. To generate the UAS-Mask-ANK and UAS-Mask-KH-Only transgenes, the KpnI (8001) site was used to separate the Mask encoding region into an N- and a C-terminal fragment, each was then used to generate the pUAST-Mask-ANK and pUAST-Mask-KH-Only respectively. All transgenic fly lines were generated by BestGene Inc. (Chino Hills, CA, USA).

Glucose and TAG measurement

Five male or female flies were collected and snap frozen in liquid nitrogen. The flies were homogenized in 200μL lysis buffer containing 1XPBS + 0.05% Triton X-100, centrifuged at 1,000×g for 1 min, and the supernatant was used to measure the protein, glucose and triacylglyceride (TAG) concentrations. 5 μL homogenate was used for TAG measurement using the Infinity Trigly-cerides reagent (Thermo Scientific). The sample was added to warmed reagent, mixed briefly, incubated at room temperature for 5 minutes, and read at 520 nm. For glucose measurement, 10μL homogenate was used to mix with 150 μL warmed Infinity Glucose

reagent (Thermo Scientific), incubate for 30 min at 37 °C, and read at 340nm. For protein measurement, 2μL homogenate was used to determine the protein concentration using DC detection kit (BioRad). All reactions were read with SpectraMAX 190 (Molecular Device).

Fly larval Neuromuscular Junction FM1-43 Dye loading

The 3rd instar wondering larvae were dissected in HL3 buffer that contains 0.43uM Ca^{2+} and then incubated in Jan's high K^+ buffer (90mM K^+, 2mM Ca^{2+}) + 4uM FM1-43 dye (final concentration) for 1 minute. The FM1-43 loading was then stopped by rinsing the larvae in Ca^{2+} free HL3 buffer rapidly twice and then slowly on a shaker for 5 minutes. The larvae were then fixed in 4% PFA (in 1X PBS) for 18 minutes, rapidly rinsed in 1X PBS twice and then incubated in HRP-Cy5 (1:1,000) for 15 minutes. The larvae then were rapidly rinsed with PBS twice and followed by a slow rinse by shaking for 5 minutes. After the rinses, the larvae were incubated in 70% glycerol (in 1X PBS) for 15 minutes and mounted for confocal imaging analysis.

Immunocytochemistry

Third instar larvae were dissected in ice-cold PBS and fixed in 4% PFA for 30 min. The fixed tissues were stained following standard procedures. Alexa-Fluor-647-conjugated goat anti-HRP at 1:1000 was used to highlight the neuronal processes.

Confocal imaging and analysis

Single-layer or z-stack confocal images were captured on a Nikon (Tokyo, Japan) C1 confocal microscope. Images shown in the same figure were acquired using the same gain from samples that had been simultaneously fixed and stained. For quantification of YFP and FM1-43 signals at the NMJs, z-stack confocal images of microtubule in larval muscle 6/7 in segment A2 were analyzed double-blinded, IMARIS software (Bitplane, Inc) was used to quantify average signal intensity at the nerve terminals.

Fly in vivo assays

The procedure of the RING assay was adapted from established protocol[7]. 10-15 male or female flies of desired genotype and age were collected in a fresh vial containing regular the day before the experiment. The next day, the flies were flipped into fresh polystyrene vials and assembled into the RING apparatus. The files then were allowed to acclimate to the environment for 15-20 minutes. The flies were then knocked down to the bottom of the vial by three sharp tap down of the RING apparatus to the surface of the bench. The flies were allowed to climb from the bottom and along the vial for 3 or 5 seconds, and a picture of the flies in the vials were taken at the end of the interval. The distance that each fly traveled during the given time interval was then measured from the image acquired. The average of the travel distance was calculated and used to represent and quantify the adult locomotor for a given condition.

qRT_PCR

20 adult fly heads were collected, rinse with PBS 3 times and then snap frozen in liquid nitrogen. The total RNA was extracted with Trizol reagent. To prepare cDNA, the RNA samples were first treated with DNase I (Invitrogen Cat#2020-08-31), and then used as template to synthesize the

cDNA using the High Capacity cDNA Revers Transcription kit (Applied Biosystem, Cat#2020-05-31). The quantitative PCR was performed with the BioRad SYBR (Cat# 1725121) and CFX96 Real-time PCR system.

Primers used in qPCR: dilp2, dilp3, dilp5 and dilp6 primers are described previously[8]; p150Glued: Forward 5'- AAGGATCTGCTTGGCACGGTT-3'; Reverse 5'-TGCCACAGTTCTCATCGCACT-3'
Primers for internal control: Rpl32: (reference: DOI: 10.1038/ncomms10368) qRpL32_f 5' -CGCACCAAGCACTTCATCCG-3'; qRpL32_r 5' -GCACGTTGTGCACCAGGAAC-3'

The results of qRT-PCR was analyzed or presented using the ΔΔCt method (Fig. S21) or the ΔCt above background method (Fig. S9-12) as described previously[9-11]. The background Ct for each primer pair was establish as the Ct of no cDNA ("water") amplification, which is normally 40. To plot "ΔC_t above background," the C_t from each gene-specific primer for each sample was normalized to all other samples by relative 18S C_t and then subtracted from the background C_t. In this way, plots are essentially Log2 scale (each cycle of amplification doubles the amount of the amplicons) and higher numbers means higher amounts of starting RNA.

Genetic Background Control

UAS-Mask line was generated in the *w^1118* background by BestGene Inc. (Chino Hills, CA, USA)[1] and was backcrossed to the *w^1118* flies (obtained from BestGene Inc.) for 5 generations. All other Mask mutant or deletion transgenes were also generated in the *w^1118* flies; and the *w^1118* flies were obtained from BestGene Inc. together with the transformants. The longevity measurement experiments with these transgenes were performed less than one year after they were established. The TH-C'-Gal4 and TH-D'-Gal4 flies were generated in the Iso[31] background[12] (obtained from Dr. Mark Wu along with the TH-C'-Gal4 and TH-D'-Gal4 flies).

Control and experimental genotypes will be generated from the same crosses to ensure equal backgrounds: either in w^{1118}/backcross background or equal heterozygous backgrounds with chromosomes contributed by w^{1118}/backcross stock (for example X-Gal4/ w^{1118} vs. X-Gal4/transgene-5x w^{1118}).

Supplemental Table 1. Summary of Genetic Backgrounds, Experiment Repeats, Results and Statistics for Lifespan Analysis.

Genotype	Genetic Crosses	Median lifespan	Percentage changes of median lifespan	P value	Number of Repeats
Figure 1					3
0273-Gal4/+	0273-Gal4 X w^{1118} (The UAS-Mask transgenic flies were generated in the w^{1118} flies.)	80	Decreased by 2.5%	P=0.96	
0273-Gal4>UAS-Mask	0273-Gal4 X UAS-Mask	78			
TH-C'-Gal4/+	TH-C'-Gal4 X	64	Increased	P<0.001	

	w^{1118} (The UAS-Mask transgenic flies were generated in the w^{1118} flies.)		by 50%		
TH-C’-Gal4/UAS-Mask	TH-C’-Gal4 X UAS-Mask	96			
TH-D’-Gal4/+	TH-D’-Gal4 X w^{1118} (The UAS-Mask transgenic flies were generated in the w^{1118} flies.)	63	Increased by 36.5%	P<0.001	
TH-D’-Gal4/UAS-Mask	TH-D’-Gal4 X UAS-Mask	86			
Figure 2					1
TH-C’>Ds	TH-C’-Gal4 X DREADDs	Male 87 day no drug; 98 day 1uM CNO	Increased by 12.6%	P=0.0013	

		Female 104 day no drug; 108 day 1uM CNO	Increased by 12.5%	P=0.0086	
TH-D’>Ds	TH-D’-Gal4 X DREADDs	Male 73 day no drug; 95 day 1uM CNO	Increased by 30.1%	P<0.001	
		Female 67 day no drug; 107 day 1uM CNO	Increased by 57%	P<0.001	
Figure 3					1
ddc^{27}/+ ; TH-C’-Gal4/+	TH-C’-Gal4 X *ddc*^{27}/CyO	76 day	Increased by 6.5%	P=0.3	
ddc^{27}/+ ; TH-C’-Gal4/UAS-Mask	TH-C’-Gal4 X *ddc*^{27}/CyO; UAS-Mask/Tm6B	81 day			

dat$^{Z2-1277}$/+ ; TH-C'-Gal4/+	TH-C'-Gal4 X *dat*$^{Z2-1277}$/CyO	79 day	Increased by 3.8%	P=0.069	
dat$^{Z2-1277}$/+ ; TH-C'-Gal4/UAS-Mask	TH-C'-Gal4 X *dat*$^{Z2-1277}$/CyO; UAS-Mask/Tm6B	82 day			
TH-C'-Gal4/*Ple*^{4}	TH-C'-Gal4 X *Ple*^{4}/Tm6B	62 day	Increased by 32.2%	P<0.001	
TH-C'-Gal4/*Ple*^{4}*,* UAS-Mask	TH-C'-Gal4 X *Ple*^{4}*,* UAS-Mask/Tm6B	82 day			
vmat^{SH0459}/+ ; TH-C'-Gal4/+	TH-C'-Gal4 X *vmat*^{SH0459}/CyO	82 day	Decreased by 14.6%	P<0.001	
vmat^{SH0459}/+ ; TH-C'-Gal4/UAS-Mask	TH-C'-Gal4 X *vmat*^{SH0459}/CyO; UAS-Mask/Tm6B	70 day			
TH-D'-Gal4/*ddc*^{27}	TH-D'-Gal4 X *ddc*^{27}/CyO	73 day	Increased by 2.7%	P=0.13	
TH-D'-Gal4/*ddc*^{27} ; UAS-Mask/+	TH-D'-Gal4 X *ddc*^{27}/CyO; UAS-Mask/Tm6B	75 day			

TH-D'-Gal4/*dat*$^{Z2-1277}$	TH-D'-Gal4 X *dat*$^{Z2-1277}$/CyO	83 day	Decreased by 10.8%	P<0.001	
TH-D'-Gal4/*dat*$^{Z2-1277}$;UAS-Mask/+	TH-D'-Gal4 X *dat*$^{Z2-1277}$/CyO; UAS-Mask/Tm6B	74 day			
TH-D'-Gal4/+; *Ple*^{4}/+	TH-D'-Gal4 X *Ple*^{4}/Tm6B	59 day	Increased by 18.6%	P<0.001	
TH-D'-Gal4/+; *Ple*^{4},UAS-Mask/+	TH-D'-Gal4 X *Ple*^{4}, UAS-Mask/Tm6B	70 day			
TH-D'-Gal4/*vmat*^{SH0459}	TH-D'-Gal4 X *vmat*^{SH0459}/CyO	77 day	Decreased by 6.5%	P=0.15	
TH-D'-Gal4/*vmat*^{SH0459}; UAS-Mask/+	TH-D'-Gal4 X *vmat*^{SH0459}/CyO; UAS-Mask/Tm6B	72 day			
Figure 4					1
UAS-TH/+	UAS-TH X Iso^{31} (The TH-C'- and TH-D'-Gal4 drivers were generated	Male 66 day; Female 108 day			

	in the Iso[31] flies.)				
TH-C'-Gal4>UAS-TH	TH-C'-Gal4 X UAS-Mask	Male 62 day; Female 103 day	Male decreased by 6.1%; Female decreased by 4.6%	Male P=0.19; Female P=0.21	
TH-D'-Gal4>UAS-TH	TH-D'-Gal4 X UAS-Mask	Male 63 day; Female 96 day	Male decreased by 4.5%; Female decreased by 11.1%	Male P=0.52; Female P<0.001	
UAS-Ddc/+	UAS-Ddc X Iso[31] (The TH-C'- and TH-D'-Gal4 drivers were generated in the Iso[31] flies.)	Male 73 day; Female 99 day			
TH-C'-Gal4>UAS-Ddc	TH-C'-Gal4 X UAS-Ddc	Male 68 day;	Male decreased	Male P=0.24;	

		Female 106 day	by 6.8%; Female increased by 7.1%	Female P<0.001	
TH-D'-Gal4>UAS-Ddc	TH-D'-Gal4 X UAS-Ddc	Male 74 day; Female 102 day	Male increased by 1.4%; Female increased by 3.0%	Male P=0.31; Female P=0.66	
Figure 7					1
TH-C'/+	TH-C'-Gal4 X *w*^{1118} (All the UAS-Mask transgenic flies were generated in the *w*^{1118} flies by the same facility.)	Male 56 Day			
		Female 80 Day			
TH-C'/UAS-Mask	TH-C'-Gal4 X UAS-Mask	Male 91 Day			
		Female 102 Day			

TH-C'/UAS-Mask-KH-Mut	TH-C'-Gal4 X UAS-Mask-KH-Mut	Male 91 Day		P=0.97 vs. TH-C'/UAS-Mask	
		Female 100 Day		P=0.83 vs. TH-C'/UAS-Mask	
TH-C'/UAS-ANK	TH-C'-Gal4 X UAS-ANK	Male 87 Day		P=0.1 vs. TH-C'/UAS-Mask	
		Female 96 Day		P=0.52 vs. TH-C'/UAS-Mask	
TH-C'/UAS-KH Only	TH-C'-Gal4 X UAS- KH Only	Male 74 Day		P<0.01 vs. TH-C'/UAS-Mask	
		Female 75 Day		P<0.01 vs. TH-C'/UAS-	

				Mask	
UAS-GFP-UNC104/+	UAS-GFP-UNC104 X Iso^{31} (The TH-C'- and TH-D'-Gal4 drivers were generated in the Iso^{31} flies.)	68 Day			
UAS-GFP-UNC104/+; TH-C'/+	UAS-GFP-UNC104 X TH-C'-Gal4	95 Day	Increased by 39.7%	P<0.001	
TH-D'/+; UAS-GFP-UNC104	UAS-GFP-UNC104 X TH-D'-Gal4	98 Day	Increased by 44.1%	P<0.001	
UAS-$P150^{Glued}$ RNAi/+	UAS-$P150^{Glued}$ RNAi X Iso^{31} (The TH-C'- and TH-D'-Gal4 drivers were generated in the Iso^{31} flies.)	88 day			

UAS-P150^{Glued} RNAi /+ ;; TH-C'/+	UAS-P150^{Glued} RNAi X TH-C'-Gal4	101 Day	Increased by 14.8%	P<0.001	
UAS-P150^{Glued} RNAi /+ ; TH-D'/+	UAS-P150^{Glued} RNAi X TH-D'-Gal4	94 day	Increased by 6.8%	P=0.28	

SI Figure Legends

Figure S1. Overexpressing Mask in dopaminergic neurons extends lifespan in flies.

A) Survivorship curves of control flies (Ddc-Gal4 > UAS-tdTomato), flies expressing *mask* RNAi (Ddc-Gal4 > Mask RNAi) or wild type Mask transgenes in the DA neurons (Ddc-Gal4 >UAS- Mask). Knocking down Mask in the DA neurons does not impact the lifespan in the flies, while overexpressing Mask profoundly extend the lifespan in flies. B) Survivorship curves of control flies (Elav-Gal4 > UAS-tdTomato), flies expressing *mask* RNAi in all neurons (Elav-Gal4 > Mask RNAi), or flies overexpressing Mask in all neurons (Elav-Gal4 > UAS-Mask). Loss-of-function of Mask in all neurons leads to partial lethality during the development (data not shown). Those that do eclose show significantly reduced lifespan. In contrast to the effect of *mask* loss-of-function, overexpressing Mask in all neurons does not affect lifespan in flies.

Figure S2. The lifespan extension induced by Mask overexpression is specifically mediated by dopaminergic neurons.

Survivorship curves of male flies that overexpress Mask in A) all cells (Da-Gal4, ubiquitous driver); B) glial cells (Repo-Gal4); C) all neurons (elav-Gal4); or dopaminergic neurons D) Ddc-Gal4 and E) TH-Gal4. Only when Mask is overexpressed in dopaminergic neurons, the lifespan of the flies is profoundly prolonged. Overexpressing Mask ubiquitously in the entire body or in all neurons does not affect the longevity. Overexpressing Mask in glial cells only moderately extends the lifespan in flies.

Figure S3. Overexpressing Mask in cholinergic neurons moderately shortens lifespan in flies.

The Survivorship curves of male and female flies overexpressing Mask in cholinergic neurons driven by Cha-Gal4. A) Female flies show moderatly reduced lifespan, while lifespan in B) male flies are not affected.

Figure S4. Suppressing the Neuronal Activation of the TH-C' or TH-D' Neurons Does not impact the Lifespan in Flies.

The Survivorship curves of flies expressing the engineered DREADD-Di ($G_{\alpha i}$) in the (A, B) TH-C' or (C, D) TH-D' DANs are shown. The flies are raised on food containing DMSO only (No Drug), 1uM, 10uM or 100uM Clozapine N-oxide (CNO) for their entire adulthood. Inhibiting the activation of the TH-C' or TH-D' DANs does not affect the lifespan in flies.

Figure S5. Chronically activating the TH-C' and TH-D' dopaminergic neurons in the adult flies moderately shortens lifespan.

Survivorship curves of male A) and female B) flies expressing the thermal sensitive cation channel TrpA1 in the TH-C' or the TH-D' DANs. The activation of these neurons were achieved by raising the flies at 29°C shortly after eclosion. Long-term activation of the TH-C' neurons leads to 33% reduction in the lifespan (median lifespan shortened from 44 days to 33 days.), and activating the TH-D' neurons results in 10% reduction in the lifespan (median lifespan shortened from 31 days to 28 days.). No effect on lifespan was detected in females when TH-C' or TH-D' dopaminergic neurons were activated by TrpA1.

Figure S6. Ubiquitously Elevating dopamine activity is not sufficient to extend lifespan in flies. Survivorship curves of A) female and B) male w^{1118} flies raised on regular food, or on food

containing 1mg/ml L-DOPA; and C) female and D) male w^{1118} flies raised on regular food, or on food containing 75uM of SKF-82958, 30uM of Quinpirole, or both drugs are shown. None of the treatments extended the lifespan of male flies. Feeding female flies with dopamine precursor L-DOPA moderately shortened the lifespan (median lifespan reduced from 88 days in the control flies to 78 days in L-DOPA fed flies, ~12% reduction). Neither dopamine receptor agonists alone nor together shows significant effects on female lifespan, although a small population of fed flies showed weaker tolerance and died earlier than untreated group. Log-Rank Test *p* values: C) p = 0.25 (w^{1118}+SKF-82958 vs. w^{1118}), p = 0.091 (w^{1118}+Quinpirole vs. w^{1118}), p = 0.20 (w^{1118}+SKF-82958+Quinpirole vs. w^{1118}); and D) p = 0.68 (w^{1118}+SKF-82958 vs. w^{1118}), p = 0.99 (w^{1118}+Quinpirole vs. w^{1118}), p = 0.29 (w^{1118}+SKF-82958+Quinpirole vs. w^{1118}).

Figure S7. Overexpressing Mask in the TH-C' or TH-D' Neurons Does not Change Food Intake in Flies.

The food intake in a 24 hour interval was measured using a Capillary Feeder assay (CAFE). The amount of food consumed was represented by the distance (mm) between the positions of the liquid food surface at the beginning and the end of the experiments. Food intake of flies that overexpress Mask in the TH-C' neurons is compared to the control flies at A) 10-day or B) 30-day old ages. Food intake of flies that overexpress Mask in the TH-D' neurons is compared to the control flies at C) 10-day or D) 30-day old ages. No differences in food intake was detected between the long-lived flies and the control flies. n=3 for all sample groups.

Figure S8. Overexpressing Mask in the TH-C' or TH-D' Neurons moderately increase TAG and Glucose levels at mid and late ages in Flies.

A, B) Age related whole body Glucose/Protein and C, D) TAG/Protein levels in Mask overexpressing or control flies. Mask overexpressing flies contain higher glucose and TAG levels at mid (30-day) and late (50-day) ages, with the exception that female flies overexpressing Mask in the TH-C' neurons show similar levels of TAG compared to the controls. n=4 for all sample groups.

Figure S9. Overexpressing Mask in the TH-C' or TH-D' DANs does not lead to consistent changes in Dilp2 transcript levels in the adult brains.

The transcript levels of Dilp2 in the adult brain was measured in newly eclosed and 2-, 5-, 7-, 10-, 15-, 30- and 50-day old flies. A) Male flies overexpressing Mask in the TH-C' neurons show reduced Dilp2 levels at 7- and 15-day old ages. B) Male flies overexpressing Mask in the TH-D' neurons show reduced Dilp2 levels at 50-day old age. C) Female flies overexpressing Mask in the TH-C' neurons show reduced Dilp2 levels at 10-day old age. D) Female flies overexpressing Mask in the TH-D' neurons show no changes in Dilp2 levels in adulthood. Rpl32 was used as the internal reference gene for the qRT-PCR analysis. *** $P < 0.05$

Figure S10. Overexpressing Mask in the TH-C' or TH-D' DA neurons does not lead to consistent changes in Dilp3 transcript levels in the adult brains.

The transcript levels of Dilp3 in the adult brain was measured in newly eclosed and 2-, 5-, 7-, 10-, 15-, 30- and 50-day old flies. A) Male flies overexpressing Mask in the TH-C' neurons show no changes in Dilp3 levels. B) Male flies overexpressing Mask in the TH-D' neurons show reduced Dilp3 levels at 50-day old age. C) Newly eclosed female flies overexpressing Mask in the TH-C'

neurons show reduced Dilp3 levels. D) Female flies overexpressing Mask in the TH-D’ neurons show reduced Dilp3 levels at 50-day old age. Rpl32 was used as the internal reference gene for the qRT-PCR analysis. *** $P < 0.05$

Figure S11. Overexpressing Mask in the TH-C’ or TH-D’ DA neurons does not lead to consistent changes in Dilp5 transcript levels in the adult brains.

The transcript levels of Dilp5 in the adult brain was measured in newly eclosed and 2-, 5-, 7-, 10-, 15-, 30- and 50-day old flies. A) Male flies overexpressing Mask in the TH-C’ neurons show reduced Dilp5 level at 15-day old age. B) Male flies overexpressing Mask in the TH-D’ neurons show reduced Dilp5 levels at 15- and 50-day old ages. C) Female flies overexpressing Mask in the TH-C’ neurons show no changes in Dilp5 levels. D) Female flies overexpressing Mask in the TH-D’ neurons show no changes in Dilp5 levels. Rpl32 was used as the internal reference gene for the qRT-PCR analysis. *** $P < 0.05$

Figure S12. Overexpressing Mask in the TH-C’ or TH-D’ DA neurons does not lead to consistent changes in Dilp6 transcript levels in the adult brains.

The transcript levels of Dilp6 in the adult brain was measured in newly eclosed and 2-, 5-, 7-, 10-, 15-, 30- and 50-day old flies. A) Male flies overexpressing Mask in the TH-C’ neurons show no changes in Dilp6 levels. B) Male flies overexpressing Mask in the TH-D’ neurons show increased Dilp6 level at 30-day old age. At 50-day old age, Dilp6 transcript level in the head is moderately reduced. C) Newly eclosed female flies overexpressing Mask in the TH-C’ neurons show reduced Dilp6 level. D) Female flies overexpressing Mask in the TH-D’ neurons show

reduced Dilp6 level at 50-day old age. Rpl32 was used as the internal reference gene for the qRT-PCR analysis. *** $P < 0.05$

Figure S13. The KH domain in the Mask protein is required for Mask to promote autophagy.

(A) Confocal images of GFP-mCherry auto-fluorescence (using the same gain) from fixed larval muscles in control animals (24B-Gal4>UAS-GFP-mCherry-ATG8), animals overexpressing wild type Mask protein (24B-Gal4>UAS-GFP-mCherry-ATG8+UAS-Mask) or Mask-KH-Mut protein (24B-Gal4>UAS-GFP-mCherry-ATG8+UAS-Mask-KH-Mut). (B) Quantification of mCherry intensity in the larval body wall muscles 6 is shown. Overexpressing Mask in the muscle promotes autophagy, while the KH-Mutant protein fail to induce the same effects.

Figure S14. Expressing Mask in neurons in the adult flies fail to induce lifespan extension compared to ATG1.

A, D) Wild type flies and flies B, E) expressing ATG1 (GS-Elav>UAS-ATG1) or C, F) Mask (GS-Elav>UAS-Mask) in their neurons are raised on regular food or food containing 50ug/ml RU488 in order to assess the effects of adulthood neuronal expression of Mask or ATG1 on longevity. GS-Elav induces pan-neuronal expression of transgenes in the presence of RU488. 50ug/ml RU488 induces a moderate adverse effect on lifespan in wild type flies. Expressing ATG1 in neurons leads to moderate lifespan extension (in reference to wild type flies). The lifespan of Mask overexpressing flies, to the contrary, show no difference compared to the non-expressing flies.

Figure S15. Overexpressing Mask Promotes formation of YFP-Rab5-Positive Membrane Structures at the Presynaptic Terminal at fly larval neuromuscular junctions (NMJs).

A) Representative confocal images of synapses in control (elav-Gal4>*mask* control RNAi), *mask* knockdown (elav-Gal4>*mask* RNAi) and Mask overexpressing (elav-Gal4>UAS-Mask) larvae are highlighted with HRP (red). YFP auto-fluorescence (using the same gain) from fixed sample marks the YFP-Rab5-positive compartments at the nerve terminals. B) Quantification of the intensity of the YFP signals in the nerve terminal of muscle 6/7 are shown. Knocking down Mask significantly reduces, while overexpressing Mask, increases the intensity of the YFP-Rab5-positetive compartments.

Figure S16. Mask Promotes FM1-43 Dye Loading at Fly Larval Neuromuscular Junctions (NMJs).

A) Representative synapses and boutons in control (elav-Gal4/+) and Mask overexpressing (elav-Gal4 > UAS-Mask) larvae are highlighted with HRP (blue). FM1-43 dye loaded in the vesicle at the nerve terminals is shown in red. B) Quantification of FM1-43 dye intensity in the NMJs on muscle 6/7.

The membrane nonpermeable dye FM1-43 can be taken up into the synaptic vesicles through endocytosis, allowing the quantification of vesicle exocytosis-endocytosis events taking place at the nerve terminals. After stimulation, significantly higher level of FM1-43 loading was detected in presynaptic boutons in Mask overexpressing synapses compared to the controls. No significant FM1-43 loading at the NMJs was detected in a 30 minute interval at rest state in

control or Mask overexpressing synapses (data not shown), indicating that overexpressing Mask does not affect endocytosis per se, but rather, the activity-dependent vesicle fusion events.

Figure S17. Enhancing endocytosis and vesicle trafficking in the TH-C' neurons moderately Extend Lifespan.

Survivorship curves of flies expressing the wild type Rab5 protein (UAS-YFP-Rab5), the dominant negative form of Rab5 (UAS-YFP-Rab5-S43N), or the constitutively active form of Rab5 protein (UAS-YFP-Rab5-Q88L) are shown. A, B) Overexpressing the wild type Rab5 protein, or C, D) the dominant negative form of Rab5 in the TH-C' neurons does not affect lifespan in flies. E) Overexpressing the constitutively active form of Rab5 protein in the TH-C' neurons moderately extends lifespan in male flies. The median lifespan increased from 68 days in the control flies to 74 days in TH-C'-Gal4 > UAS-YFP-Rab5-Q88L flies, ~8.8% increase. F) Overexpressing the constitutively active form of Rab5 protein in the TH-C' neurons does not affect the lifespan in female flies.

Figure 18. TH-C' and TH-D' DANs together do not further extend lifespan.

Survivorship curves of A) male or B) female flies of control or flies overexpressing Mask protein in both the TH-C' and TH-D' dopaminergic neurons.

Figure 19. The TH-C'- and TH-D'-Gal4 Drivers show minimal non-TH expression in the brain.

Representative z-projections of confocal imaging. Adult brains of flies that express GAP-GFP in A) the TH-C' or B) the TH-D' dopaminergic neurons are co-stained with anti-GFP (green) and anti-Tyrosine Hydroxylase (TH, red) antibodies. Both the TH-C'- and the TH-D'-Gal4 show minimal and distinct non-TH expression in the brain (arrow heads). Co-expression of GFP and TH were confirmed in optic z-sections taken along the dorsal-ventral axis of the entire central brain.

Figure 20. Knocking down *mask* in the TH-C' or TH-D' DANs does not extend lifespan in flies.

Survivorship curves of A) male or B) female flies of control or flies expressing *mask* RNAi transgene in the TH-C' or the TH-D' DANs. Knocking down *mask* in the TH-C' DANs does not lead to statistically significant alterations in lifespan; while knocking down *mask* in the TH-D' DANs moderately shortens lifespan in both male and female flies.

Figure 21. Validation of genetic manipulations in flies.

A) Western blot of Mask on 3rd instar larval muscle homogenates from 24B-Gal4 driven UAS-Td-tomato (control), *mask* RNAi (knockdown) or UAS-Mask (overexpression) animals. (B) UAS-GFP-Unc104 expression in control (Elav/+) or larval brain (Elav>GFP-Unc104). 3rd instar larval brains were dissected and fixed with 4%PFA and stained with anti-GFP antibody. C) P150Glued expression levels in the adult brain were quantified by quantitative RT-PCR (qRT-PCR). The weak RNAi of P150Glued reduced to about 80% compared to the control flies (Elav>UAS-Vector). Rpl32 was used as the internal reference gene for qRT-PCR analysis.

References

1. Zhu, M., Li, X., Tian, X. & Wu, C. Mask loss-of-function rescues mitochondrial impairment and muscle degeneration of Drosophila pink1 and parkin mutants. *Hum Mol Genet* **24**, 3272-3285 (2015). PubMed PMID: 25743185
2. Budnik, V., Koh, Y. H., Guan, B., Hartmann, B., Hough, C., Woods, D. & Gorczyca, M. Regulation of synapse structure and function by the Drosophila tumor suppressor gene dlg. *Neuron* **17**, 627-640 (1996). PubMed PMID: 8893021
3. Kayser, M. S., Yue, Z. & Sehgal, A. A critical period of sleep for development of courtship circuitry and behavior in Drosophila. *Science* **344**, 269-274 (2014). PubMed PMID: 24744368
4. Zhang, M. Z., Yao, B., Wang, S., Fan, X., Wu, G., Yang, H., Yin, H., Yang, S. & Harris, R. C. Intrarenal dopamine deficiency leads to hypertension and decreased longevity in mice. *J Clin Invest* **121**, 2845-2854 (2011). PubMed PMID: 21701066
5. Wiemerslage, L., Schultz, B. J., Ganguly, A. & Lee, D. Selective degeneration of dopaminergic neurons by MPP(+) and its rescue by D2 autoreceptors in Drosophila primary culture. *J Neurochem* **126**, 529-540 (2013). PubMed PMID: 23452092
6. Walters, M. R., Dutertre, M. & Smith, C. L. SKF-82958 is a subtype-selective estrogen receptor-alpha (ERalpha) agonist that induces functional interactions between ERalpha and AP-1. *J Biol Chem* **277**, 1669-1679 (2002). PubMed PMID: 11700319
7. Nichols, C. D., Becnel, J. & Pandey, U. B. Methods to assay Drosophila behavior. *J Vis Exp* (2012). PubMed PMID: 22433384
8. Matsuda, H., Yamada, T., Yoshida, M. & Nishimura, T. Flies without trehalose. *J Biol Chem* **290**, 1244-1255 (2015). PubMed PMID: 25451929
9. Bredemeyer, A. J., Geahlen, J. H., Weis, V. G., Huh, W. J., Zinselmeyer, B. H., Srivatsan, S., Miller, M. J., Shaw, A. S. & Mills, J. C. The gastric epithelial progenitor cell niche and differentiation of the zymogenic (chief) cell lineage. *Dev Biol* **325**, 211-224 (2009). PubMed PMID: 19013146
10. Tian, X., Jin, R. U., Bredemeyer, A. J., Oates, E. J., Błazewska, K. M., McKenna, C. E. & Mills, J. C. RAB26 and RAB3D are direct transcriptional targets of MIST1 that regulate exocrine granule maturation. *Mol Cell Biol* **30**, 1269-1284 (2010). PubMed PMID: 20038531
11. Capoccia, B. J., Lennerz, J. K., Bredemeyer, A. J., Klco, J. M., Frater, J. L. & Mills, J. C. Transcription factor MIST1 in terminal differentiation of mouse and human plasma cells. *Physiol Genomics* **43**, 174-186 (2011). PubMed PMID: 21098683
12. Liu, Q., Liu, S., Kodama, L., Driscoll, M. R. & Wu, M. N. Two dopaminergic neurons signal to the dorsal fan-shaped body to promote wakefulness in Drosophila. *Curr Biol* **22**, 2114-2123 (2012). PubMed PMID: 23022067

Figure S1

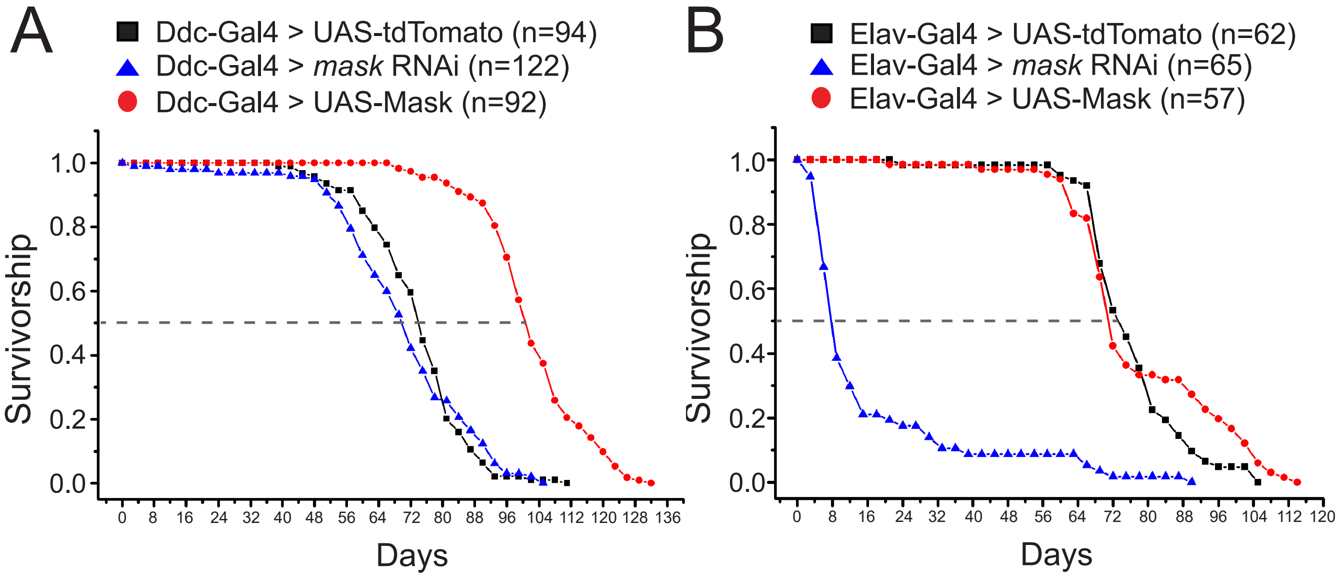

p < 0.001 Control vs UAS-Mask

p = 0.40 Control vs RNAi

p < 0.001 Control vs RNAi

p = 0.16 Control vs UAS-Mask

Figure S2

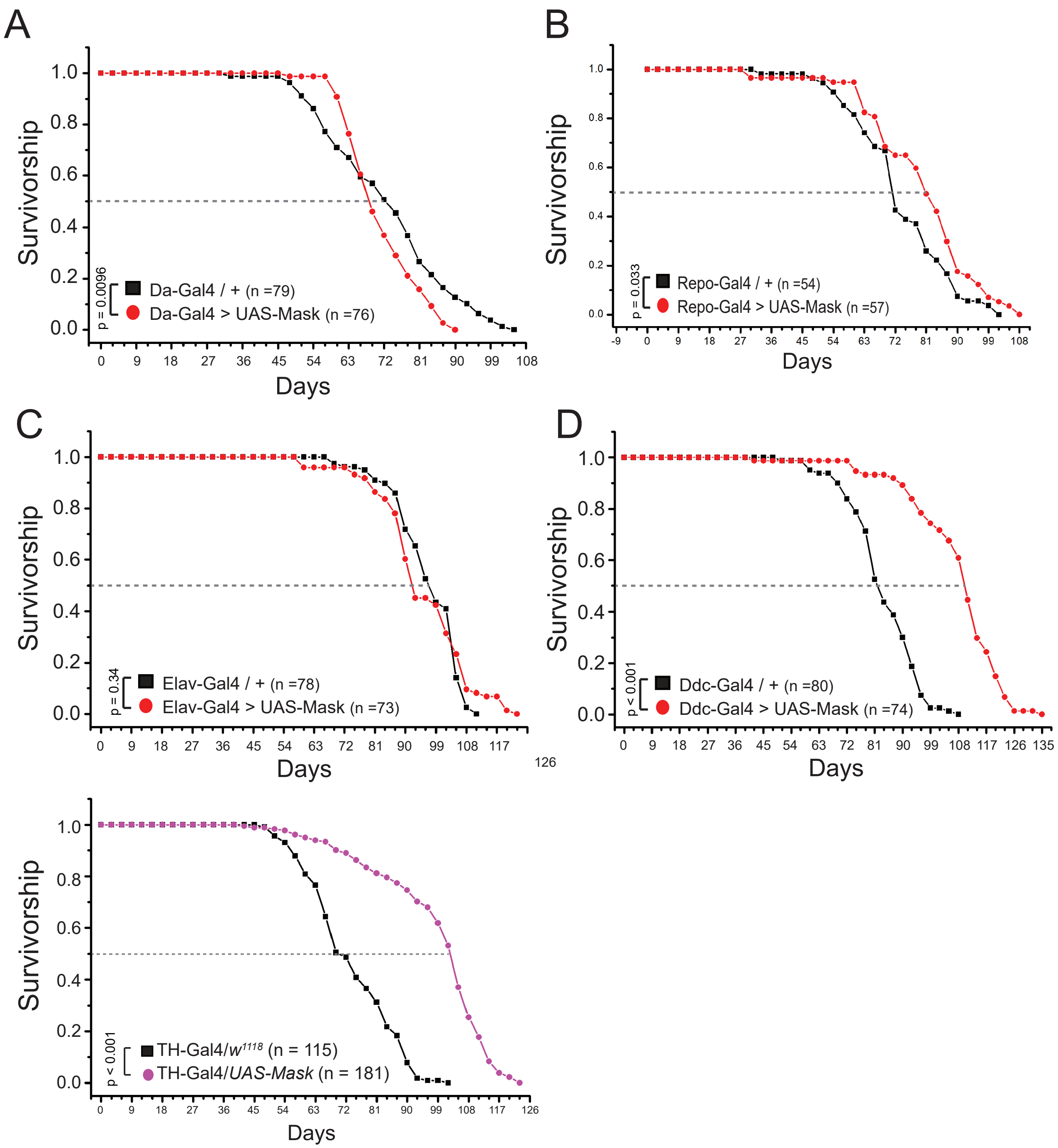
A
Survivorship
Days
p = 0.0096
Da-Gal4 / + (n =79)
Da-Gal4 > UAS-Mask (n =76)
B
Survivorship
Days
p = 0.033
Repo-Gal4 / + (n =54)
Repo-Gal4 > UAS-Mask (n =57)
C
Survivorship
Days
p = 0.34
Elav-Gal4 / + (n =78)
Elav-Gal4 > UAS-Mask (n =73)
D
Survivorship
Days
p < 0.001
Ddc-Gal4 / + (n =80)
Ddc-Gal4 > UAS-Mask (n =74)
Survivorship
Days
p < 0.001
TH-Gal4/w1118 (n = 115)
TH-Gal4/UAS-Mask (n = 181)

Figure S3

A

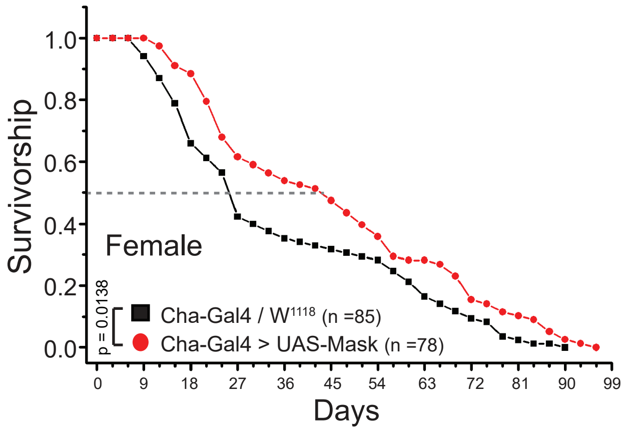
Survivorship
1.0
0.8
0.6
0.4
0.2
0.0
Female
p = 0.0138
Cha-Gal4 / W1118 (n =85)
Cha-Gal4 > UAS-Mask (n =78)
0 9 18 27 36 45 54 63 72 81 90 99
Days

B

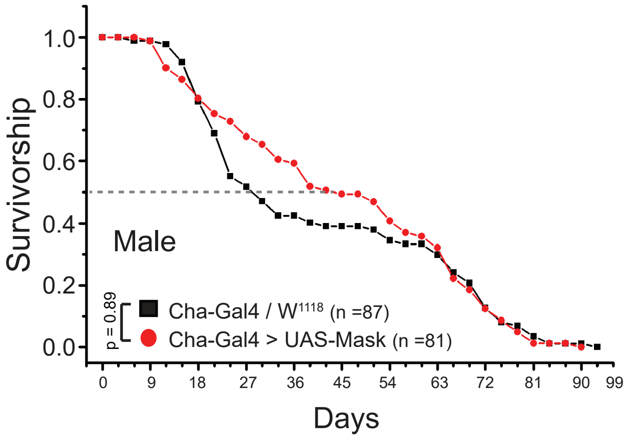
Survivorship
1.0
0.8
0.6
0.4
0.2
0.0
Male
p = 0.89
Cha-Gal4 / W1118 (n =87)
Cha-Gal4 > UAS-Mask (n =81)
0 9 18 27 36 45 54 63 72 81 90 99
Days

Figure S4

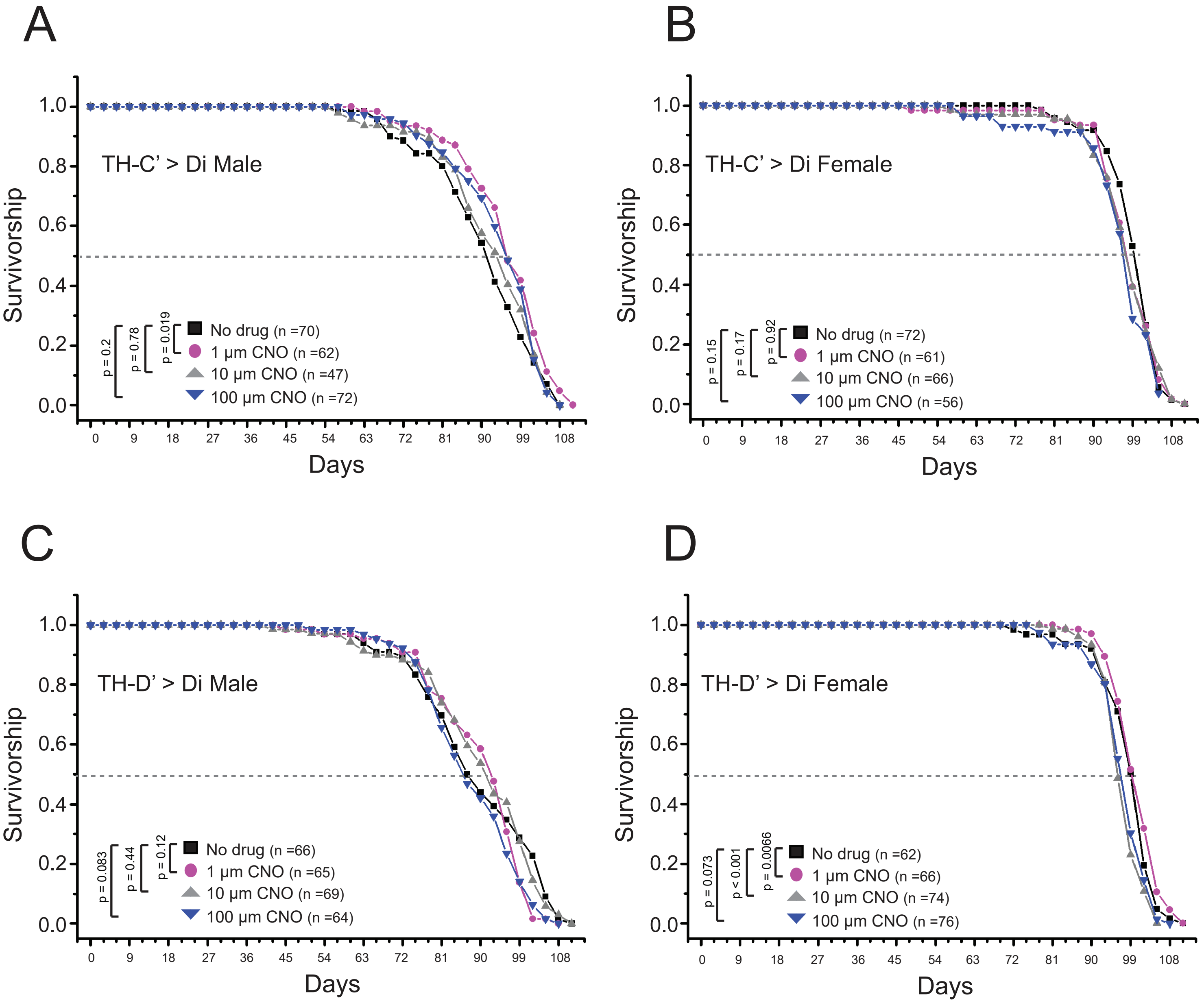

A
TH-C' > Di Male
Survivorship
Days
No drug (n =70)
1 μm CNO (n =62)
10 μm CNO (n =47)
100 μm CNO (n =72)
p = 0.2
p = 0.78
p = 0.019
B
TH-C' > Di Female
No drug (n =72)
1 μm CNO (n =61)
10 μm CNO (n =66)
100 μm CNO (n =56)
p = 0.15
p = 0.17
p = 0.92
C
TH-D' > Di Male
No drug (n =66)
1 μm CNO (n =65)
10 μm CNO (n =69)
100 μm CNO (n =64)
p = 0.083
p = 0.44
p = 0.12
D
TH-D' > Di Female
No drug (n =62)
1 μm CNO (n =66)
10 μm CNO (n =74)
100 μm CNO (n =76)
p = 0.073
p < 0.001
p = 0.0066

Figure S5

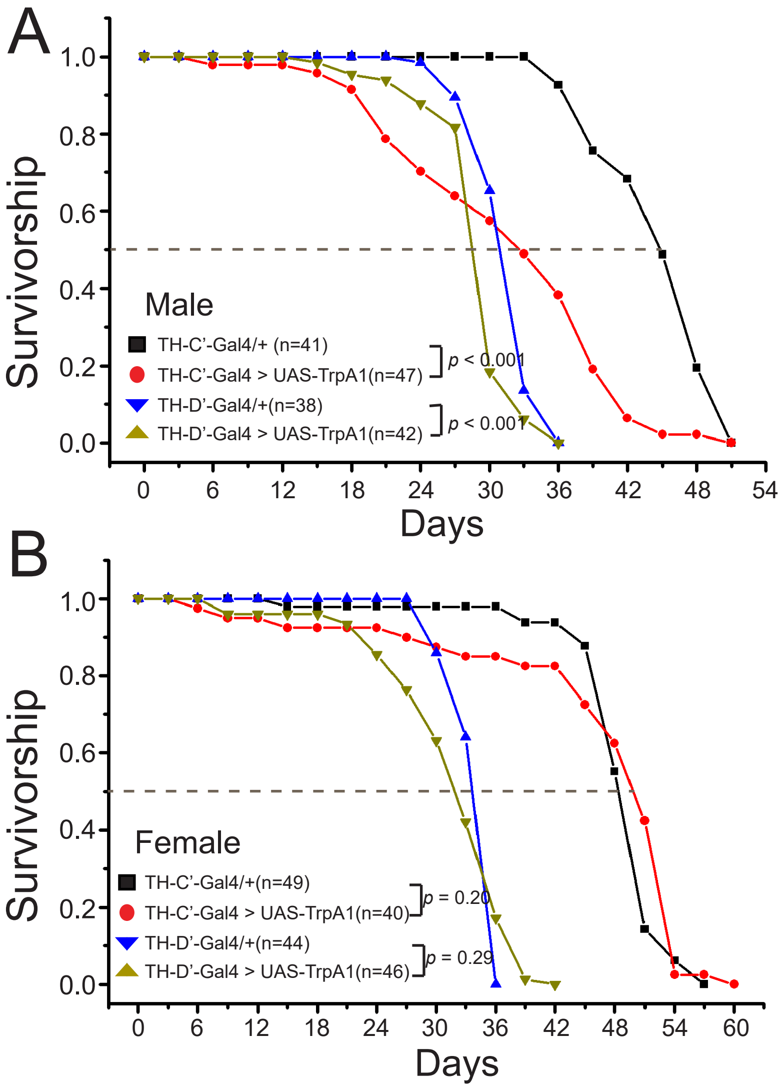

A
1.0
0.8
0.6
0.4
0.2
0.0
Survivorship
Male
TH-C'-Gal4/+ (n=41)
TH-C'-Gal4 > UAS-TrpA1(n=47)
TH-D'-Gal4/+(n=38)
TH-D'-Gal4 > UAS-TrpA1(n=42)
p < 0.001
p < 0.001
0
6
12
18
24
30
36
42
48
54
Days
B
1.0
0.8
0.6
0.4
0.2
0.0
Survivorship
Female
TH-C'-Gal4/+(n=49)
TH-C'-Gal4 > UAS-TrpA1(n=40)
TH-D'-Gal4/+(n=44)
TH-D'-Gal4 > UAS-TrpA1(n=46)
p = 0.20
p = 0.29
0
6
12
18
24
30
36
42
48
54
60
Days

Figure S6

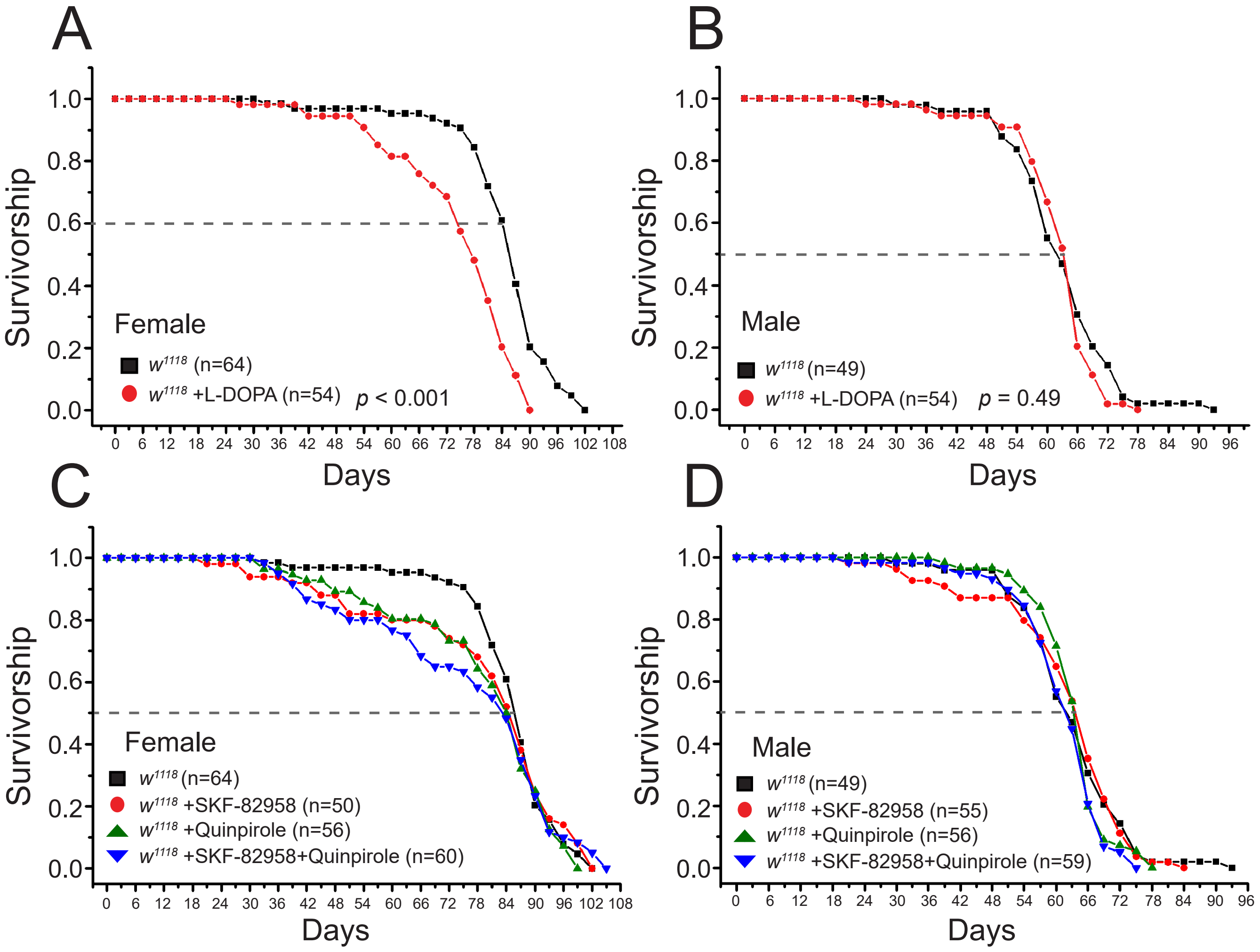
A
B
C
D
Survivorship
Days
Female
Male
w1118 (n=64)
w1118 +L-DOPA (n=54)
p < 0.001
w1118 (n=49)
w1118 +L-DOPA (n=54)
p = 0.49
w1118 +SKF-82958 (n=50)
w1118 +Quinpirole (n=56)
w1118 +SKF-82958+Quinpirole (n=60)
w1118 +SKF-82958 (n=55)
w1118 +SKF-82958+Quinpirole (n=59)

Figure S7

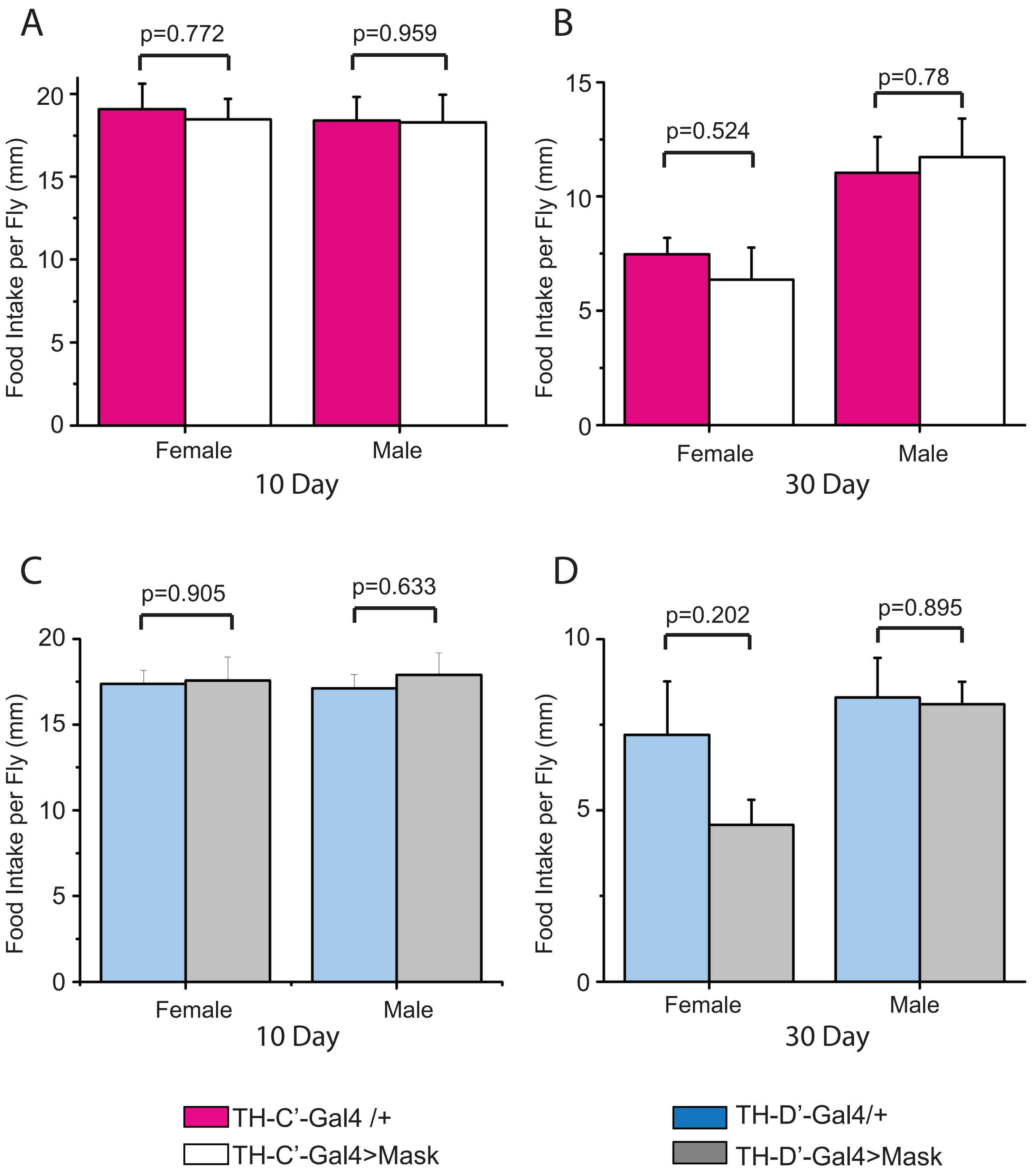

A
p=0.772
p=0.959
Food Intake per Fly (mm)
0
5
10
15
20
Female
Male
10 Day
B
p=0.524
p=0.78
Food Intake per Fly (mm)
0
5
10
15
Female
Male
30 Day
C
p=0.905
p=0.633
Food Intake per Fly (mm)
0
5
10
15
20
Female
Male
10 Day
D
p=0.202
p=0.895
Food Intake per Fly (mm)
0
5
10
Female
Male
30 Day
TH-C'-Gal4 /+
TH-C'-Gal4>Mask
TH-D'-Gal4/+
TH-D'-Gal4>Mask

Figure S8

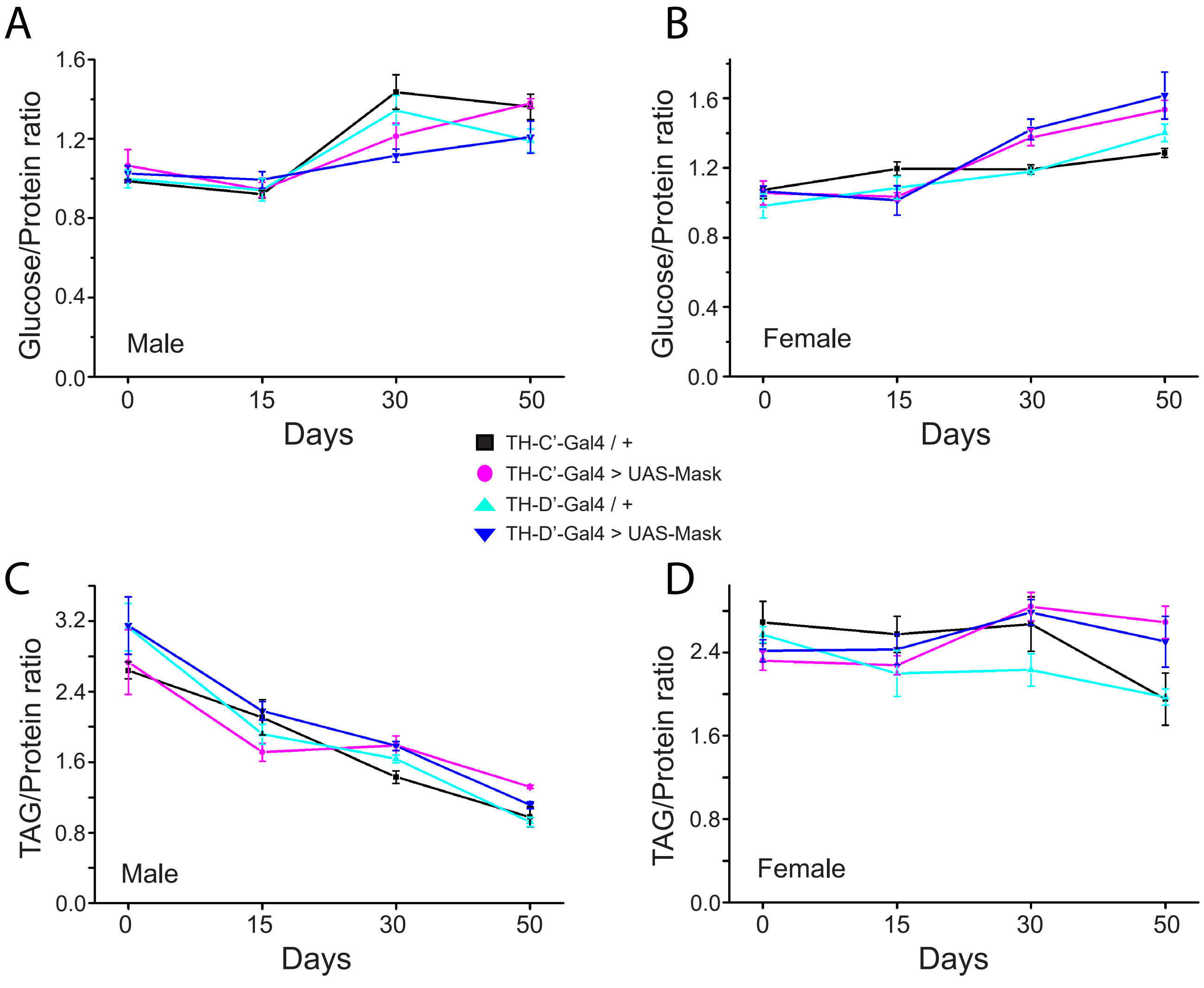
A
B
C
D
Glucose/Protein ratio
TAG/Protein ratio
Days
Male
Female
1.6
1.2
0.8
0.4
0.0
3.2
2.4
0
15
30
50
TH-C’-Gal4 / +
TH-C’-Gal4 > UAS-Mask
TH-D’-Gal4 / +
TH-D’-Gal4 > UAS-Mask

Figure S9

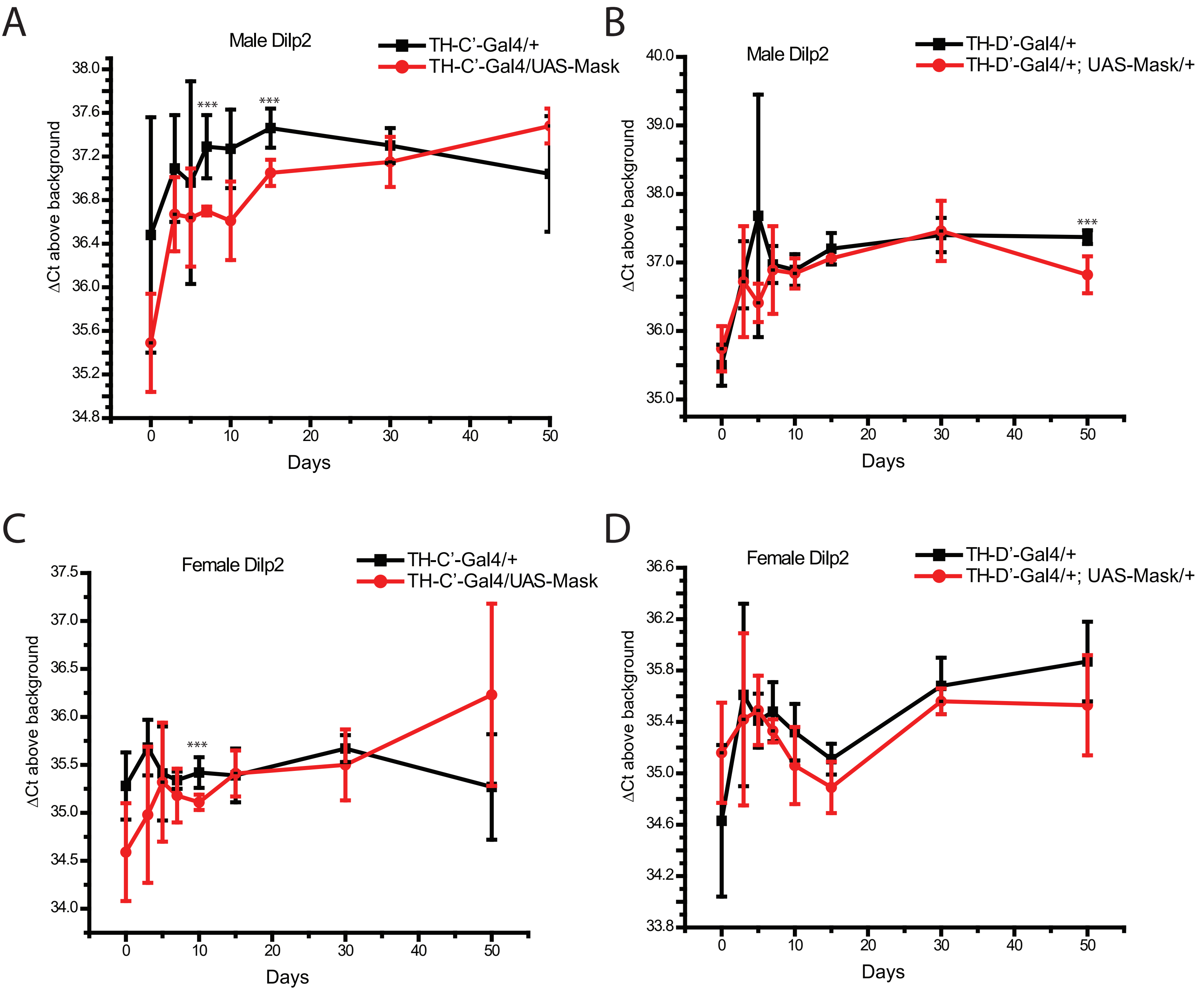

A
Male Dilp2
TH-C'-Gal4/+
TH-C'-Gal4/UAS-Mask
ΔCt above background
Days
***
B
Male Dilp2
TH-D'-Gal4/+
TH-D'-Gal4/+; UAS-Mask/+
C
Female Dilp2
TH-C'-Gal4/+
TH-C'-Gal4/UAS-Mask
D
Female Dilp2
TH-D'-Gal4/+
TH-D'-Gal4/+; UAS-Mask/+

Figure S10

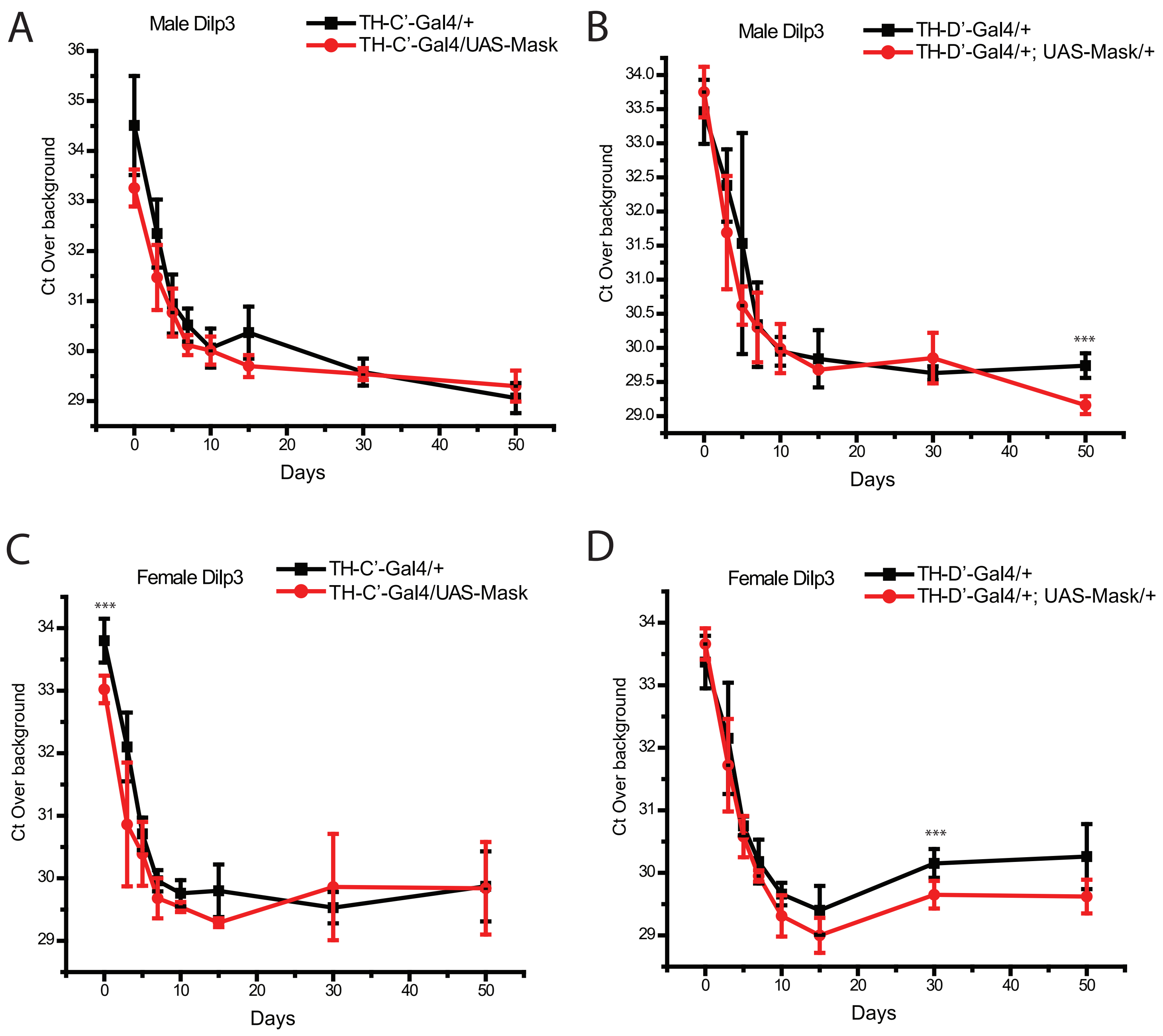
A
Male Dilp3
TH-C'-Gal4/+
TH-C'-Gal4/UAS-Mask
Ct Over background
Days
B
Male Dilp3
TH-D'-Gal4/+
TH-D'-Gal4/+; UAS-Mask/+
Ct Over background
Days
***
C
Female Dilp3
TH-C'-Gal4/+
TH-C'-Gal4/UAS-Mask
Ct Over background
Days
***
D
Female Dilp3
TH-D'-Gal4/+
TH-D'-Gal4/+; UAS-Mask/+
Ct Over background
Days
***

Figure S11

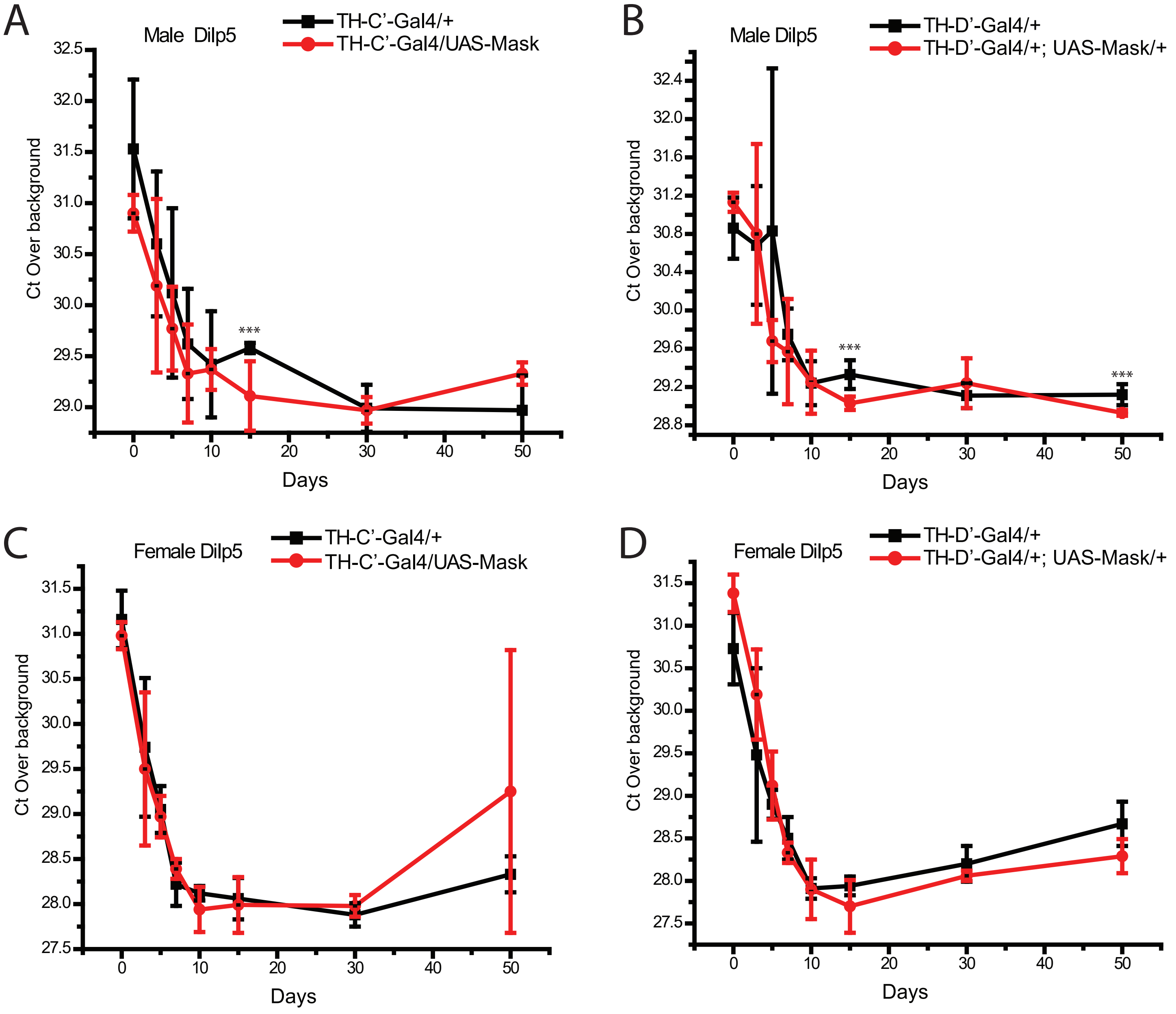
A
Male Dilp5
TH-C'-Gal4/+
TH-C'-Gal4/UAS-Mask
Ct Over background
Days
B
Male Dilp5
TH-D'-Gal4/+
TH-D'-Gal4/+; UAS-Mask/+
Ct Over background
Days
C
Female Dilp5
TH-C'-Gal4/+
TH-C'-Gal4/UAS-Mask
Ct Over background
Days
D
Female Dilp5
TH-D'-Gal4/+
TH-D'-Gal4/+; UAS-Mask/+
Ct Over background
Days

Figure S12

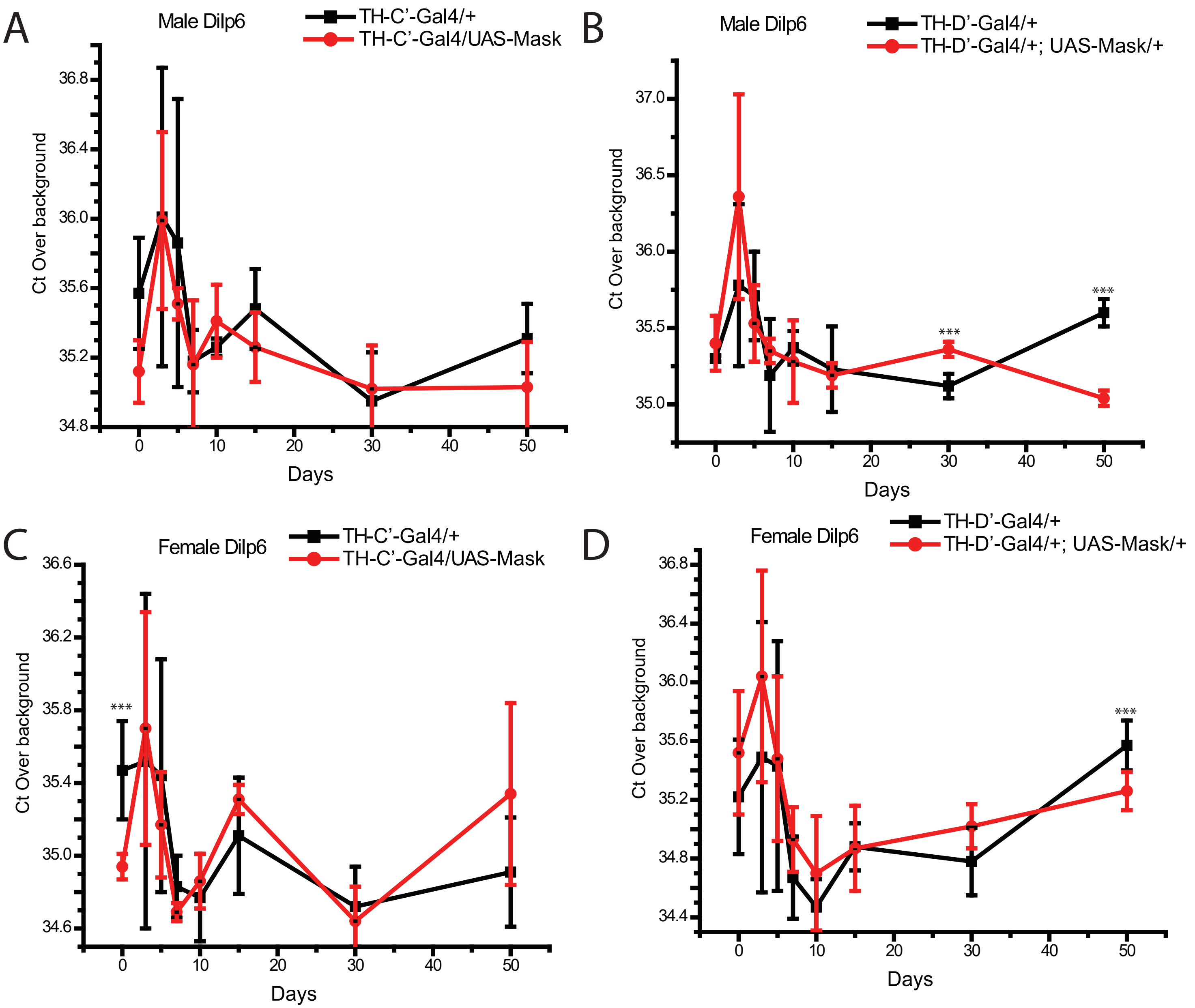

A
Male Dilp6
TH-C'-Gal4/+
TH-C'-Gal4/UAS-Mask
Ct Over background
Days
B
Male Dilp6
TH-D'-Gal4/+
TH-D'-Gal4/+; UAS-Mask/+
Ct Over background
Days
***
C
Female Dilp6
TH-C'-Gal4/+
TH-C'-Gal4/UAS-Mask
Ct Over background
Days
***
D
Female Dilp6
TH-D'-Gal4/+
TH-D'-Gal4/+; UAS-Mask/+
Ct Over background
Days
***

Figure S13

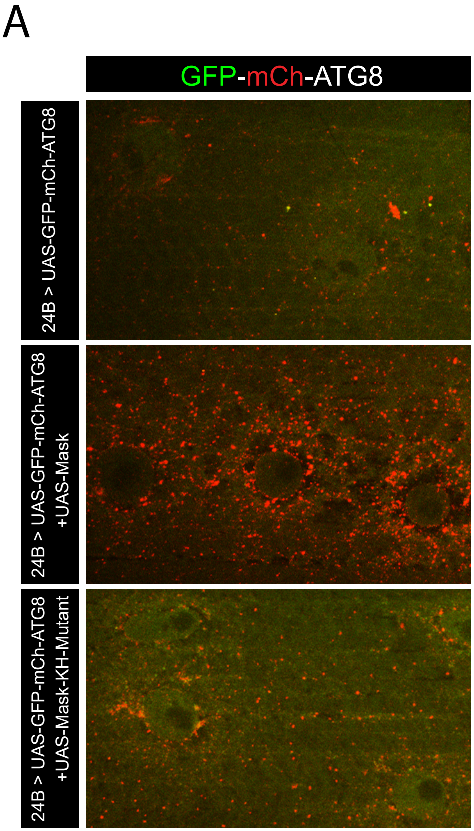

A
GFP-mCh-ATG8
24B > UAS-GFP-mCh-ATG8
24B > UAS-GFP-mCh-ATG8 +UAS-Mask
24B > UAS-GFP-mCh-ATG8 +UAS-Mask-KH-Mutant

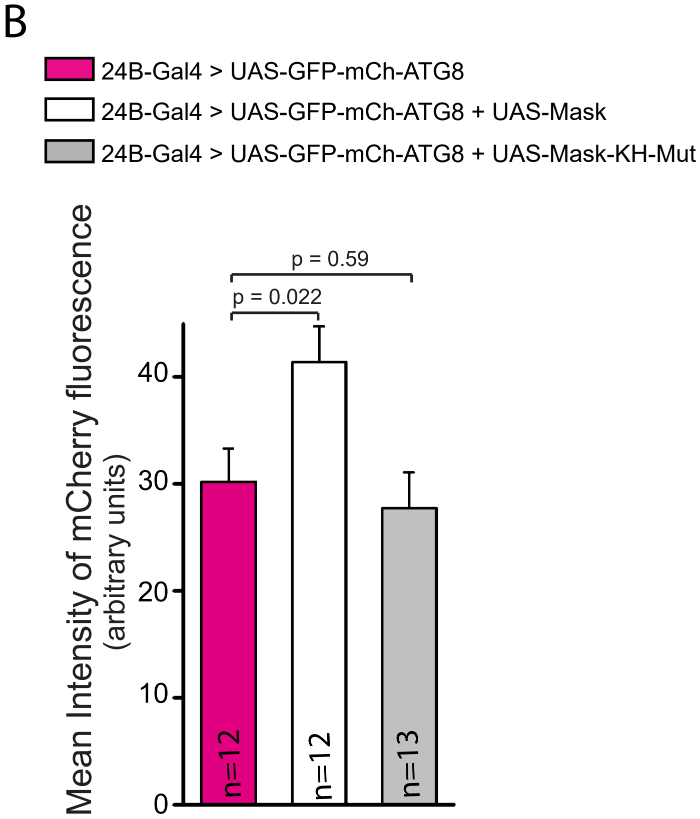

B
24B-Gal4 > UAS-GFP-mCh-ATG8
24B-Gal4 > UAS-GFP-mCh-ATG8 + UAS-Mask
24B-Gal4 > UAS-GFP-mCh-ATG8 + UAS-Mask-KH-Mut
Mean Intensity of mCherry fluorescence (arbitrary units)
p = 0.59
p = 0.022
0
10
20
30
40
n=12
n=12
n=13

Figure S14

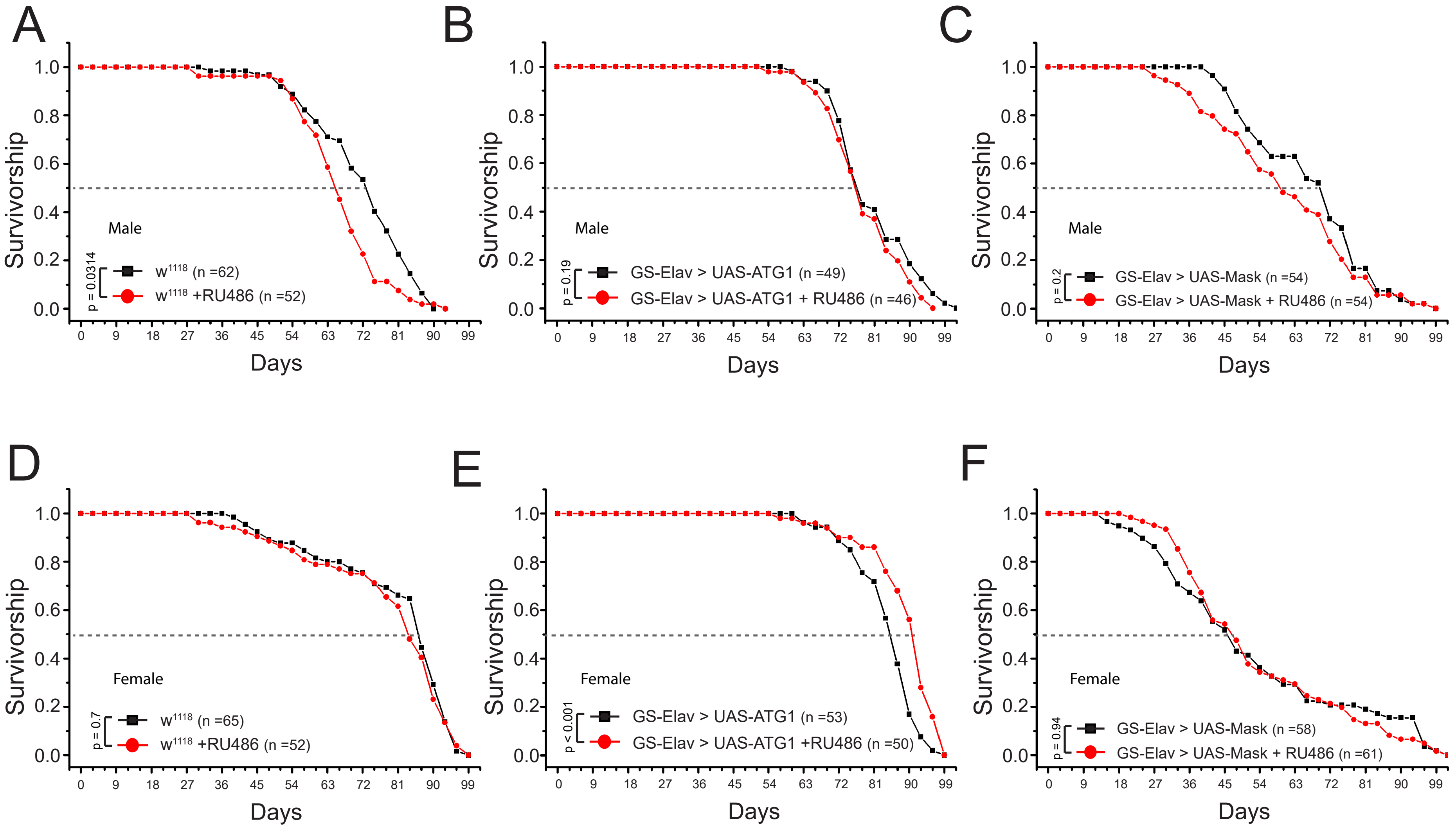

A
Survivorship
Days
Male
p = 0.0314
w1118 (n =62)
w1118 +RU486 (n =52)
B
Male
p = 0.19
GS-Elav > UAS-ATG1 (n =49)
GS-Elav > UAS-ATG1 + RU486 (n =46)
C
Male
p = 0.2
GS-Elav > UAS-Mask (n =54)
GS-Elav > UAS-Mask + RU486 (n =54)
D
Female
p = 0.7
w1118 (n =65)
w1118 +RU486 (n =52)
E
Female
p < 0.001
GS-Elav > UAS-ATG1 (n =53)
GS-Elav > UAS-ATG1 +RU486 (n =50)
F
Female
p = 0.94
GS-Elav > UAS-Mask (n =58)
GS-Elav > UAS-Mask + RU486 (n =61)

Figure S15

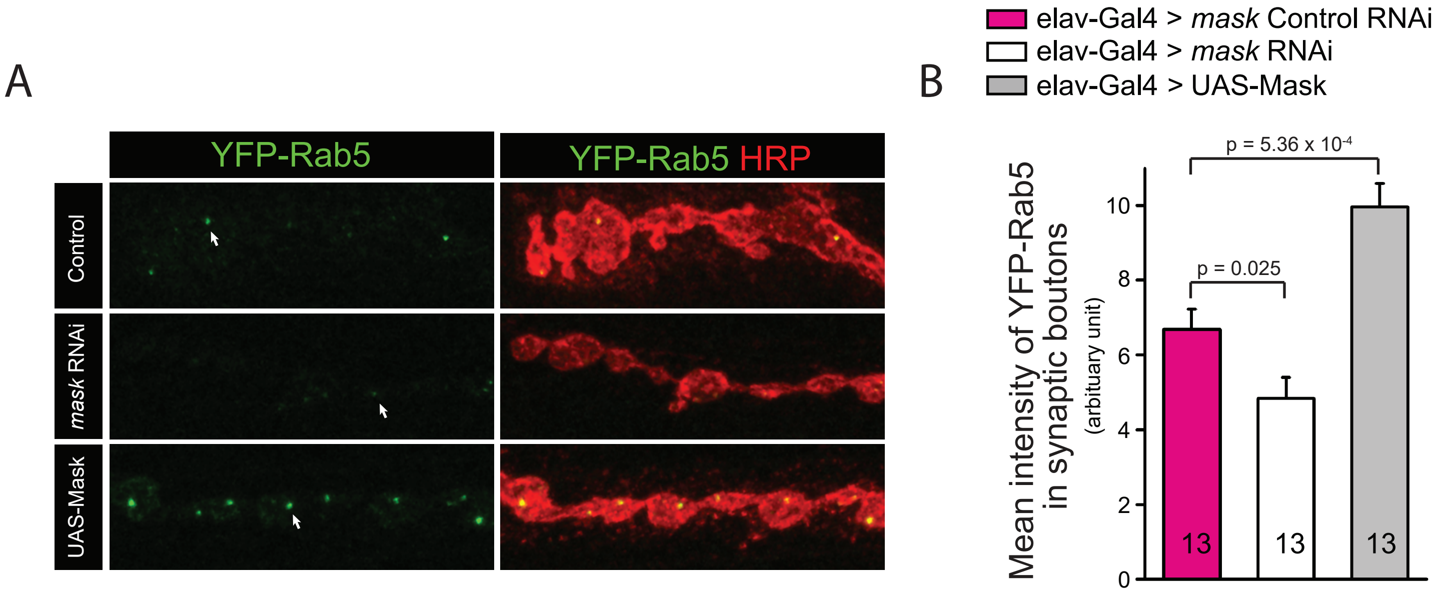
A
YFP-Rab5
YFP-Rab5 HRP
Control
mask RNAi
UAS-Mask
B
elav-Gal4 > mask Control RNAi
elav-Gal4 > mask RNAi
elav-Gal4 > UAS-Mask
p = 5.36 x 10-4
p = 0.025
Mean intensity of YFP-Rab5 in synaptic boutons
(arbituary unit)
0
2
4
6
8
10
13
13
13

Figure S16

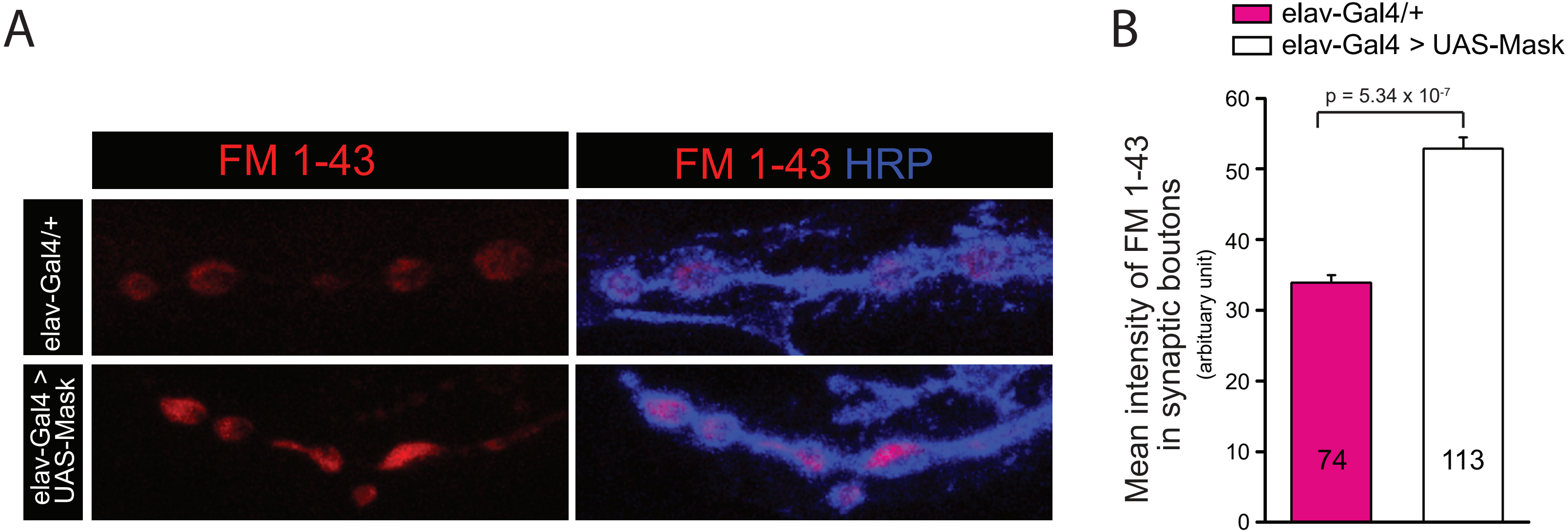
A
FM 1-43
FM 1-43 HRP
elav-Gal4/+
elav-Gal4 > UAS-Mask
B
elav-Gal4/+
elav-Gal4 > UAS-Mask
p = 5.34 x 10-7
Mean intensity of FM 1-43 in synaptic boutons
(arbituary unit)
0
10
20
30
40
50
60
74
113

Figure S17

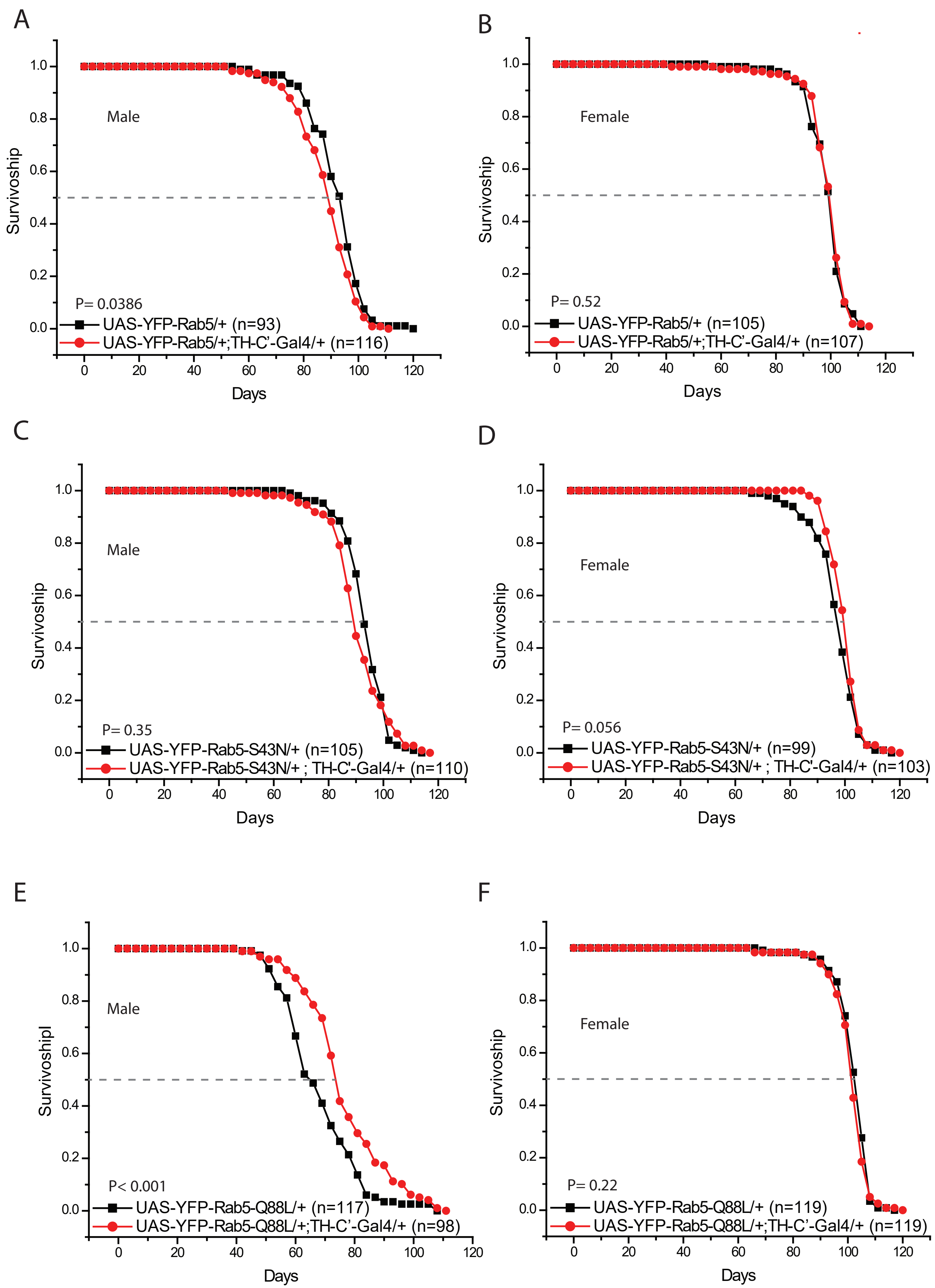
A
Male
Survivoship
Days
P= 0.0386
UAS-YFP-Rab5/+ (n=93)
UAS-YFP-Rab5/+;TH-C'-Gal4/+ (n=116)
B
Female
Survivoship
Days
P= 0.52
UAS-YFP-Rab5/+ (n=105)
UAS-YFP-Rab5/+;TH-C'-Gal4/+ (n=107)
C
Male
Survivoship
Days
P= 0.35
UAS-YFP-Rab5-S43N/+ (n=105)
UAS-YFP-Rab5-S43N/+ ; TH-C'-Gal4/+ (n=110)
D
Female
Survivoship
Days
P= 0.056
UAS-YFP-Rab5-S43N/+ (n=99)
UAS-YFP-Rab5-S43N/+ ; TH-C'-Gal4/+ (n=103)
E
Male
Survivoshipl
Days
P< 0.001
UAS-YFP-Rab5-Q88L/+ (n=117)
UAS-YFP-Rab5-Q88L/+;TH-C'-Gal4/+ (n=98)
F
Female
Survivoship
Days
P= 0.22
UAS-YFP-Rab5-Q88L/+ (n=119)
UAS-YFP-Rab5-Q88L/+;TH-C'-Gal4/+ (n=119)

Figure S18

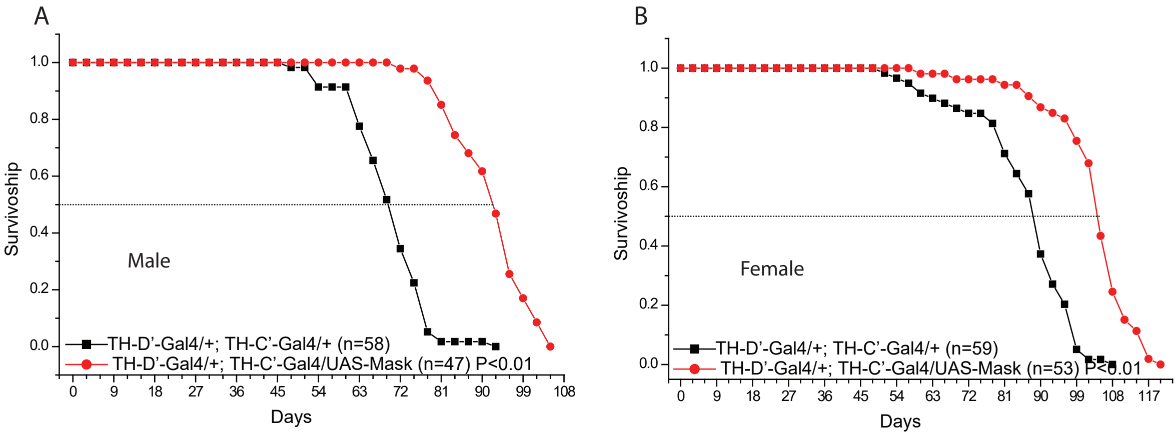
A
B
1.0
0.8
0.6
0.4
0.2
0.0
Survivoship
Days
0
9
18
27
36
45
54
63
72
81
90
99
108
117
Male
Female
TH-D'-Gal4/+; TH-C'-Gal4/+ (n=58)
TH-D'-Gal4/+; TH-C'-Gal4/UAS-Mask (n=47) P<0.01
TH-D'-Gal4/+; TH-C'-Gal4/+ (n=59)
TH-D'-Gal4/+; TH-C'-Gal4/UAS-Mask (n=53) P<0.01

Figure S19

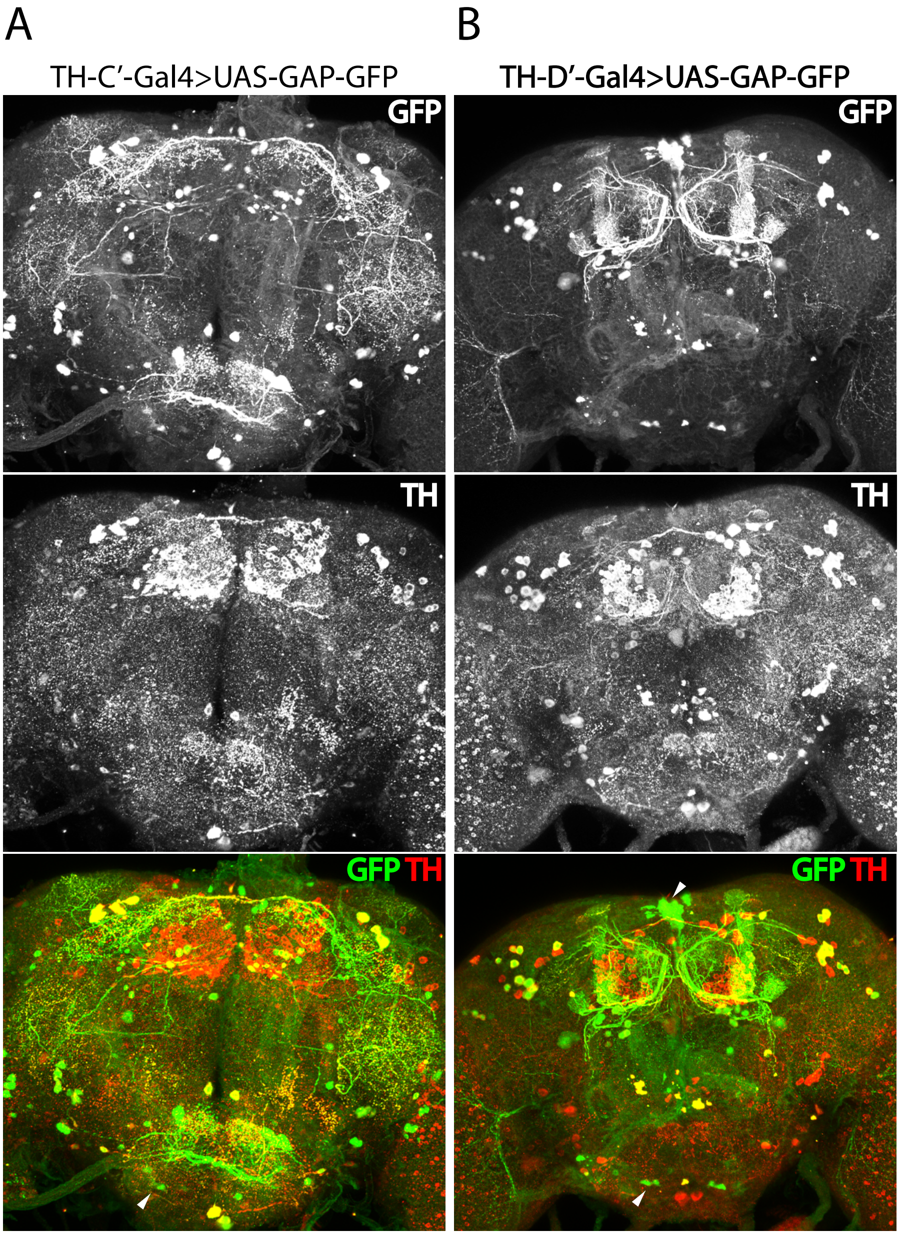
A
B
TH-C′-Gal4>UAS-GAP-GFP
TH-D′-Gal4>UAS-GAP-GFP
GFP
GFP
TH
TH
GFP TH
GFP TH

Figure S20

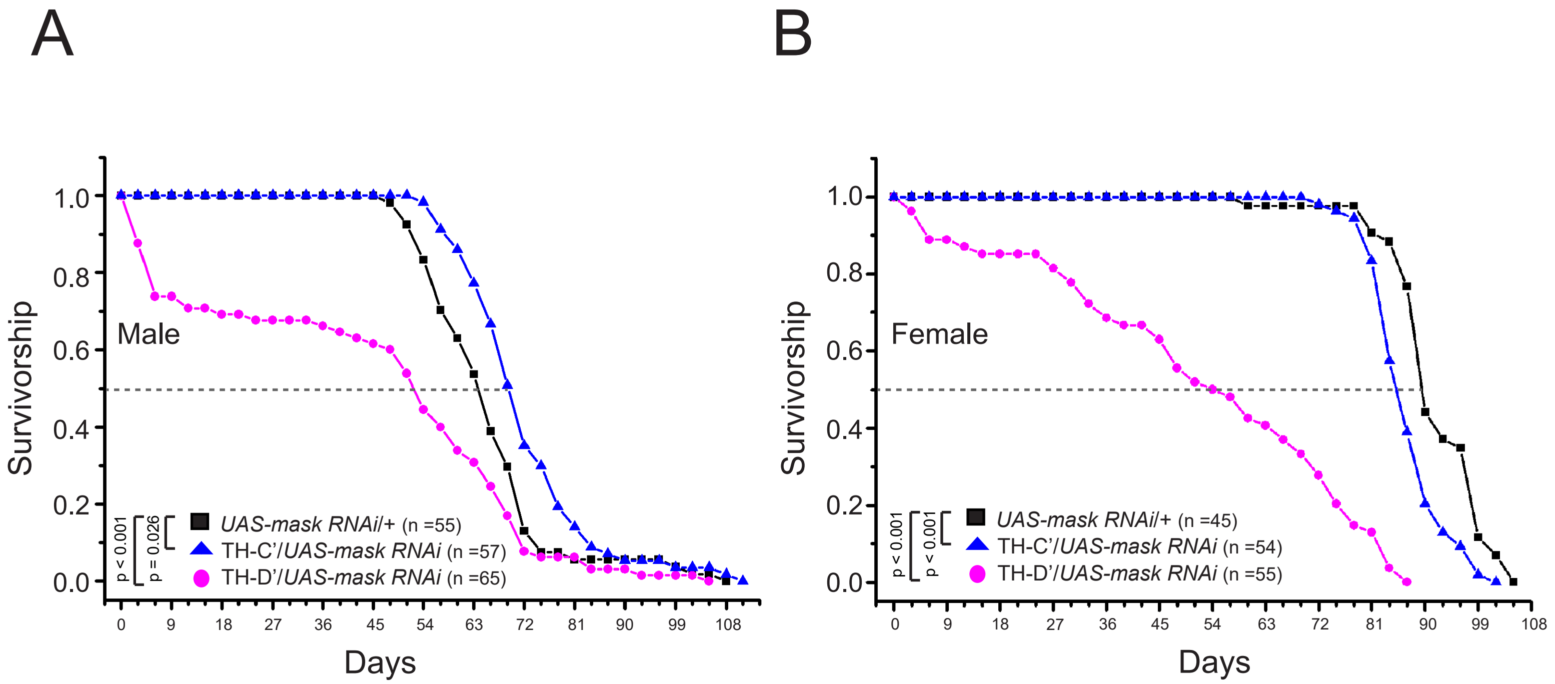
A
B
Male
Female
Survivorship
Days
1.0
0.8
0.6
0.4
0.2
0.0
0
9
18
27
36
45
54
63
72
81
90
99
108
p < 0.001
p = 0.026
UAS-mask RNAi/+ (n =55)
TH-C'/UAS-mask RNAi (n =57)
TH-D'/UAS-mask RNAi (n =65)
p < 0.001
p < 0.001
UAS-mask RNAi/+ (n =45)
TH-C'/UAS-mask RNAi (n =54)
TH-D'/UAS-mask RNAi (n =55)

Figure S21

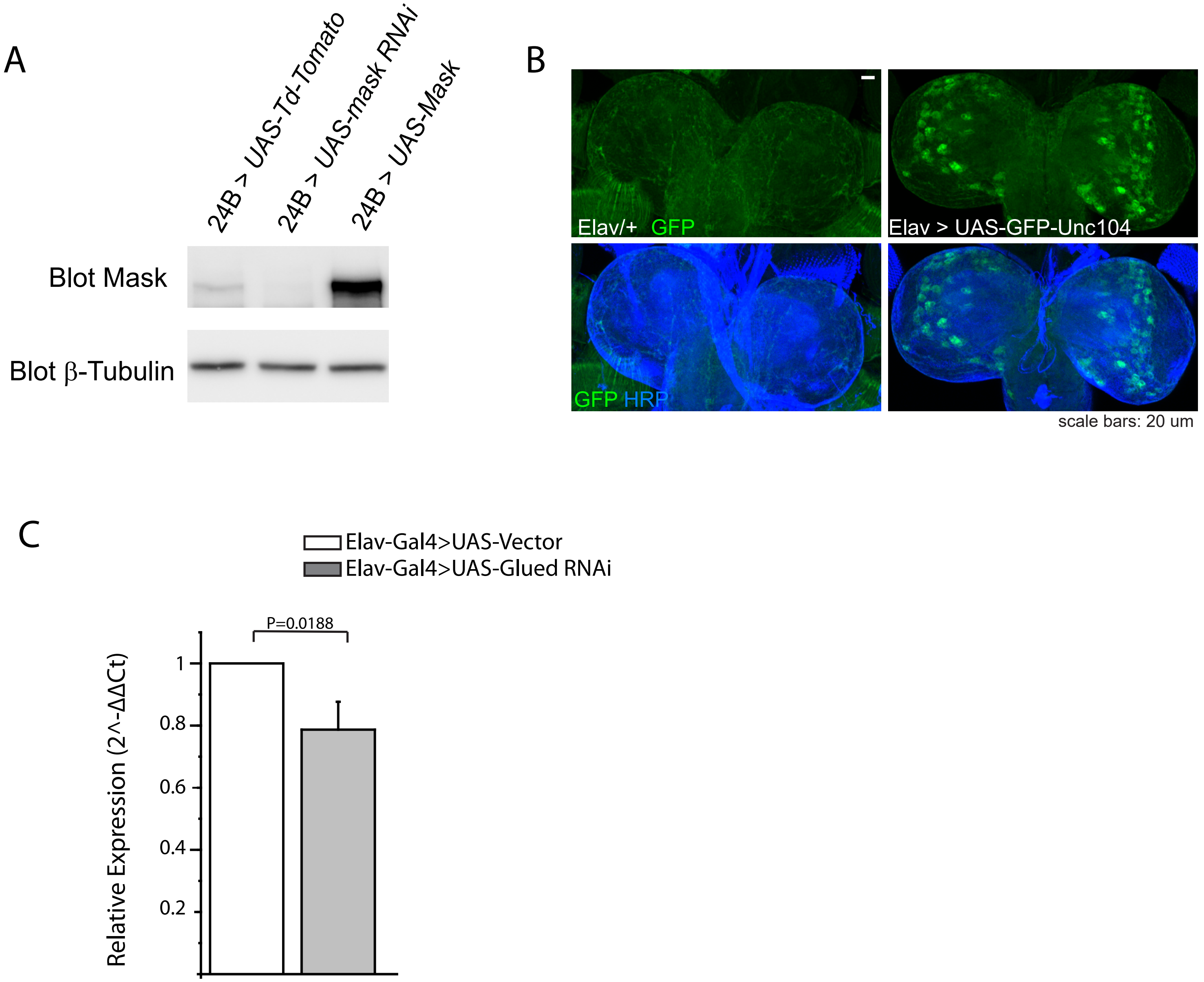
A
24B > UAS-Td-Tomato
24B > UAS-mask RNAi
24B > UAS-Mask
Blot Mask
Blot β-Tubulin
B
Elav/+ GFP
Elav > UAS-GFP-Unc104
GFP HRP
scale bars: 20 um
C
Elav-Gal4>UAS-Vector
Elav-Gal4>UAS-Glued RNAi
P=0.0188
Relative Expression (2^-ΔΔCt)
1
0.8
0.6
0.4
0.2
